# Identification of Temporal Hotspots of Distributional Change in Stress and Passive mHealth Data

**DOI:** 10.3390/s26216739

**Published:** 2026-10-24

**Authors:** Younghoon Kim, Sumanta Basu, Samprit Banerjee

**Affiliations:** 1 Department of Mathematics, University of Alabama, Tuscaloosa, AL 35487, USA; ykim124@ua.edu; 2 Department of Statistics and Data Science, Cornell University, Ithaca, NY 14853, USA; sumbose@cornell.edu; 3 Department of Population Health Sciences, Weill Cornell Medicine, New York, NY 10065, USA

**Keywords:** change point detection, moving sum, bootstrap, passive sensing, mHealth, mental health

## Abstract

Identifying temporal changes in self-reported stress and passively sensed behavioral measures is important for examining longitudinal patterns in mental health research. Conventional change point detection methods typically return isolated change points, while approaches that model relationships between stress and passive sensing variables may require explicit functional assumptions. These features can complicate the identification of potentially asynchronous distributional changes across paired series. We propose a hotspot detection framework, where hotspots are candidate temporal regions in which changes in statistical features of self-reported stress and passive sensing measures may occur at the same or nearby times. We extend the moving-sum scheme to detect changes within and across series, and construct hotspots using thresholding and bootstrap confidence-interval rules. The method does not require a specific functional relationship between series and summarizes changes as intervals rather than isolated time points. Simulation studies show that the proposed procedures achieve competitive detection rates, with performance depending on window size and the number of changes. The two hotspot rules exhibit trade-offs between retaining true change locations and constructing localized intervals. We further illustrate the method using four participants from the ALACRITY Phase I study, where the procedures identify candidate regions of potential temporal concordance between changes in daily self-reported stress and smartphone-derived behavioral measures.

## 1. Introduction

Emotional stress is the psychological strain that occurs when individuals perceive that demands exceed their coping resources. In older adults, stress may co-occur with mood disorders and may be relevant to psychotherapy and emotional well-being [1,2]. With the development of mobile health (mHealth) technologies, repeated measurements of stress and related behaviors can be collected in daily life [3,4]. Individuals can report their perceived stress through mobile devices such as smartphones and wearables, while behavioral information can be collected through passive sensing. These longitudinal measurements provide an opportunity to examine when distributional changes in self-reported stress occur and whether changes in behavioral measures occur at the same or nearby times.

Passive sensing variables are measurements collected by mHealth devices through sensors without requiring active input from the user. Typical examples include physiological measures such as heart rate variability and blood pressure, which have been used to characterize stress-related changes [5,6]. Physiological sensing measures capture autonomic or physiological responses, whereas smartphone-based behavioral measures characterize daily patterns of activity, social behavior, and sleep; the latter can be collected unobtrusively but may also depend on how consistently individuals carry and use their devices [6,7,8]. In this study, we instead focus on behavioral indicators collected through passive sensing. Patterns of physical activity, social activity, and sleep behavior provide complementary information about behavioral changes that may occur in temporal proximity to changes in self-reported stress. These behavioral measures can be collected unobtrusively in daily life using widely available mobile devices.

The goal of studying passive sensing variables in this context is to examine temporal patterns in behavioral measures alongside self-reported stress, which may later inform prediction using machine learning (ML) algorithms when self-reported measures are unavailable. However, directly applying conventional ML methods to these datasets can lead to poor performance [9,10,11]. This is because both passive sensing variables and self-reported stress may exhibit non-stationarity; their mean and variability may change across different time periods, and these local variations can become obscured when analyzed over the entire observation period.

To address this challenge, change point detection (CPD; see [12,13,14] for comprehensive surveys) can be applied as a pre-processing step to identify time points where data characteristics shift, allowing each segment to be analyzed or labeled separately before being used in ML models. In the context of stress detection from passive sensing variables, pre-defined rule-based methods have been used [15,16].

More recently, CPD algorithms have emerged as a data-driven alternative for identifying transitions in stress levels. For example, Hosseini et al. [17] applied a CPD algorithm [18] to their multimodal sensor data to identify abrupt physiological or behavioral changes associated with transitions between low- and high-stress states in nurses during their work shifts. For older patients, Kim et al. [19] developed a framework combining the CPD algorithm by [20] with a linear model linking passive sensing variables to self-reported stress, with the goal of improving the prediction of future stress states.

However, existing approaches to analyzing stress-related time series often focus on isolated change point estimates or rely on assumptions about the relationship between passive sensing variables and self-reported stress. In contrast, this study focuses on identifying temporal regions of potentially asynchronous distributional changes across the two series without explicitly modeling their relationship. Accordingly, the proposed framework characterizes potential temporal concordance between distributional changes rather than quantifying statistical association or causal relationships between the two series. This distinction is important when detected changes do not occur at exactly the same time, when multiple distributional features change simultaneously, or when the underlying association between the two series is difficult to specify. Missing the exact or nearby timing of such changes can undermine the usefulness of passive sensing variables and pose challenges for their validation as digital biomarkers in future studies [7,21].

### 1.1. Motivating Examples

**Figure 1:**
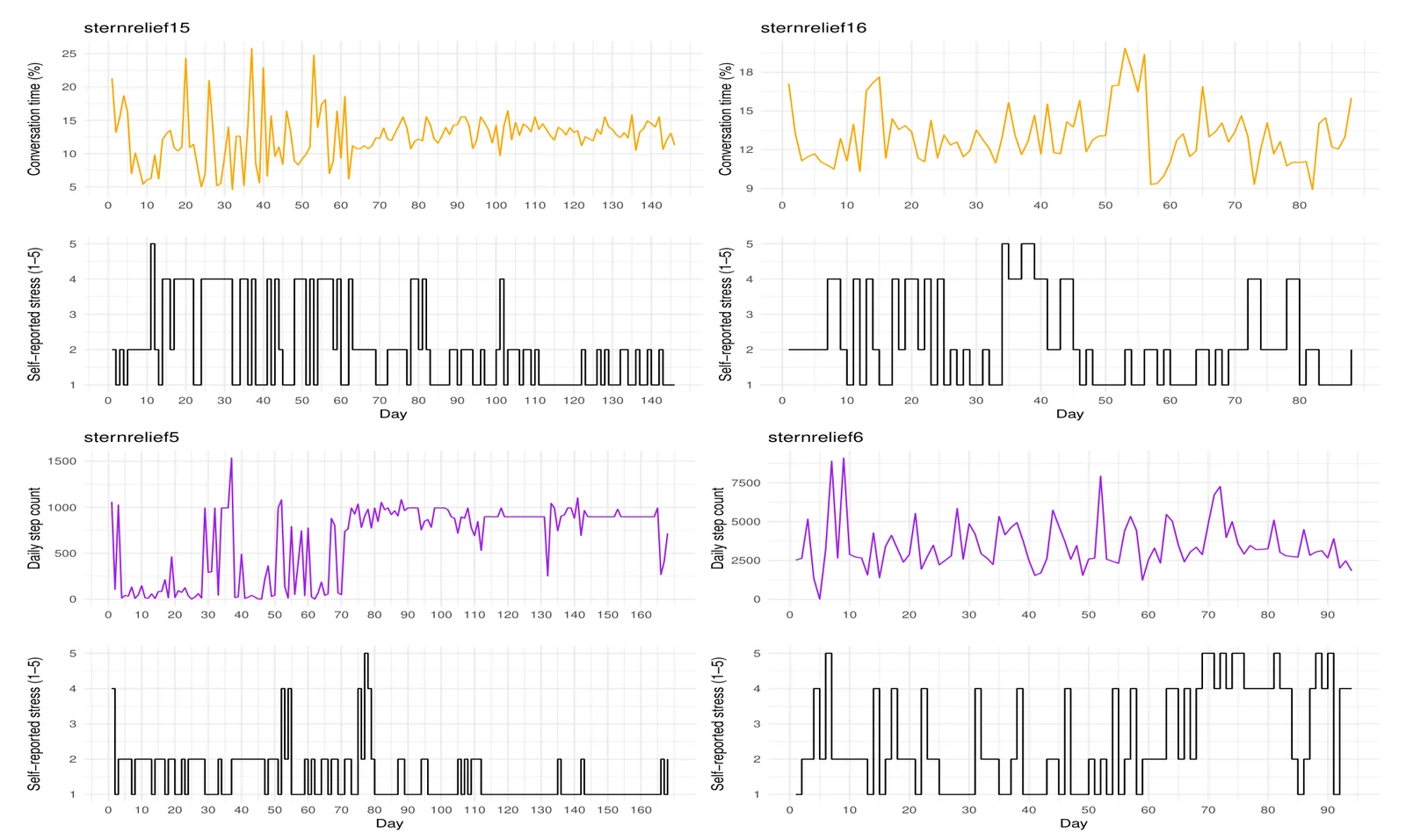
Representative patterns in passive sensing variables and self-reported stress from four patients in ALACRITY Phase I data. In each panel, the colored lines represent passive sensing variables, and the black lines represent self-reported stress.

Examples of pairs of passive sensing variables and self-reported stress from four different patients are shown in Figure 1. The details of the series are given in Section 5.4. The time plots suggest that distributional changes in the paired series may occur at nearby but different times, indicating potentially asynchronous changes across the two series. Moreover, particularly for passive sensing variables, the observed changes cannot be clearly categorized into a single type, such as changes solely in the mean or variance.

The limitations of conventional CPD algorithms can be summarized as follows. First, CPD methods targeting a single distributional feature at a time may not adequately capture changes involving multiple features, such as simultaneous shifts in the mean and variance. However, as illustrated in Figure 1, passive sensing variables and self-reported stress may exhibit changes involving multiple distributional features, including the mean and variance. This highlights the need for a more flexible framework capable of identifying changes across multiple statistical characteristics within a series. Second, conventional CPD algorithms usually report specific change points, whereas changes in paired series may occur at nearby but different times. Representing the results as temporal regions rather than isolated points can therefore accommodate potentially asynchronous changes. Third, temporal proximity between changes in passive sensing variables and self-reported stress may be informative even when no fixed functional relationship between the two series is specified. Finally, some of the changes illustrated in Figure 1 appear relatively subtle, which may further complicate change point detection.

### 1.2. Contributions

In this article, we develop a framework for identifying temporal hotspots of distributional change, focusing on changes in the mean and variance of passive sensing variables and self-reported stress. The proposed hotspots are designed to capture temporal regions in which distributional changes in self-reported stress and passive sensing variables may occur simultaneously or in temporal proximity. The framework thereby provides a tool for examining potential temporal concordance between changes in self-reported stress and behavioral measures.

This study makes several novel contributions. First, it introduces the concept of hotspots and proposes a data-driven method to identify them. This addresses a key limitation of conventional CPD algorithms, which typically detect only isolated time points rather than extended periods of change. By tracking local variations in statistical features, the algorithm can detect more candidate time points corresponding to potentially subtle distributional shifts in self-reported stress. Second, it jointly tracks changes across multiple distributional features, capturing diverse types of concurrent changes in a given series. This ensemble approach provides a more complete characterization of underlying dynamics than univariate metrics alone. Finally, the method is model-free: it does not assume a specific functional relationship between passive sensing variables and self-reported stress, while allowing distributional changes in the two series to occur at the same or nearby times. The dependence among detector components is incorporated through locally estimated covariance structures, allowing different types of distributional changes to be evaluated jointly.

### 1.3. Organization of Paper

The remainder of this article is organized as follows. Section 2 defines hotspots. Section 3 focuses on detecting change points for various types of changes. This involves the modification of the existing CPD algorithm applied in our proposed method. Section 4 describes how the initially identified change points are aggregated using ensemble strategies to generate robust change regions. The numerical experiments evaluating the performance of the method are presented in Section 5. Finally, Section 6 concludes with a discussion.

## 2. Definition of Hotspots

We define hotspots as candidate temporal regions in which distributional changes in self-reported stress and passive sensing variables may occur at the same or nearby times. The passive sensing variables considered in this study capture behavioral patterns (e.g., physical activity, social activity, and sleep). Hotspots therefore highlight potential temporal concordance between changes in the two series while allowing their timing to differ. They do not quantify statistical association, correlation, or causal relationships between self-reported stress and passive sensing variables, nor do they necessarily indicate worsening stress or clinical deterioration. Rather, they identify periods that may warrant further examination.

The proposed algorithm proceeds in two main steps. First, it identifies candidate locations of different types of distributional changes within and across the series. Second, it combines threshold-exceedance regions or confidence intervals obtained from the corresponding detectors to construct stress-anchored hotspot regions. This interval-based representation accommodates uncertainty in the timing of distributional changes as well as potentially asynchronous changes between self-reported stress and passive sensing variables.

## 3. Finding Change Points

As the first step of identifying hotspots, we find the change points where all different types of distributional shifts occur in either univariate or bivariate series.

Throughout the rest of the article, we denote a univariate passive sensing variable by $X_{t}$ and stress levels by $Y_{t}$, where $\left\{X_{t}\right\}$ and $\left\{Y_{t}\right\}$ refer to subsets or complete sets of samples of $X_{t}$ and $Y_{t}$, respectively.

### 3.1. Pre-Processing

To apply the proposed algorithm to the raw dataset, two pre-processing steps are necessary. First, we handle the discrete ordinal nature of the stress-response series by transforming them into continuous representations, enabling the use of statistical models and change point detection methods that assume continuous inputs. Second, we debias the passive sensing variables by distinguishing genuinely low behavioral activity from spuriously low measurements caused by device non-use (e.g., when the phone is turned off, not carried, or the app is inactive) and applying a bias-correction procedure based on auxiliary device-use information.

**Figure A1:**
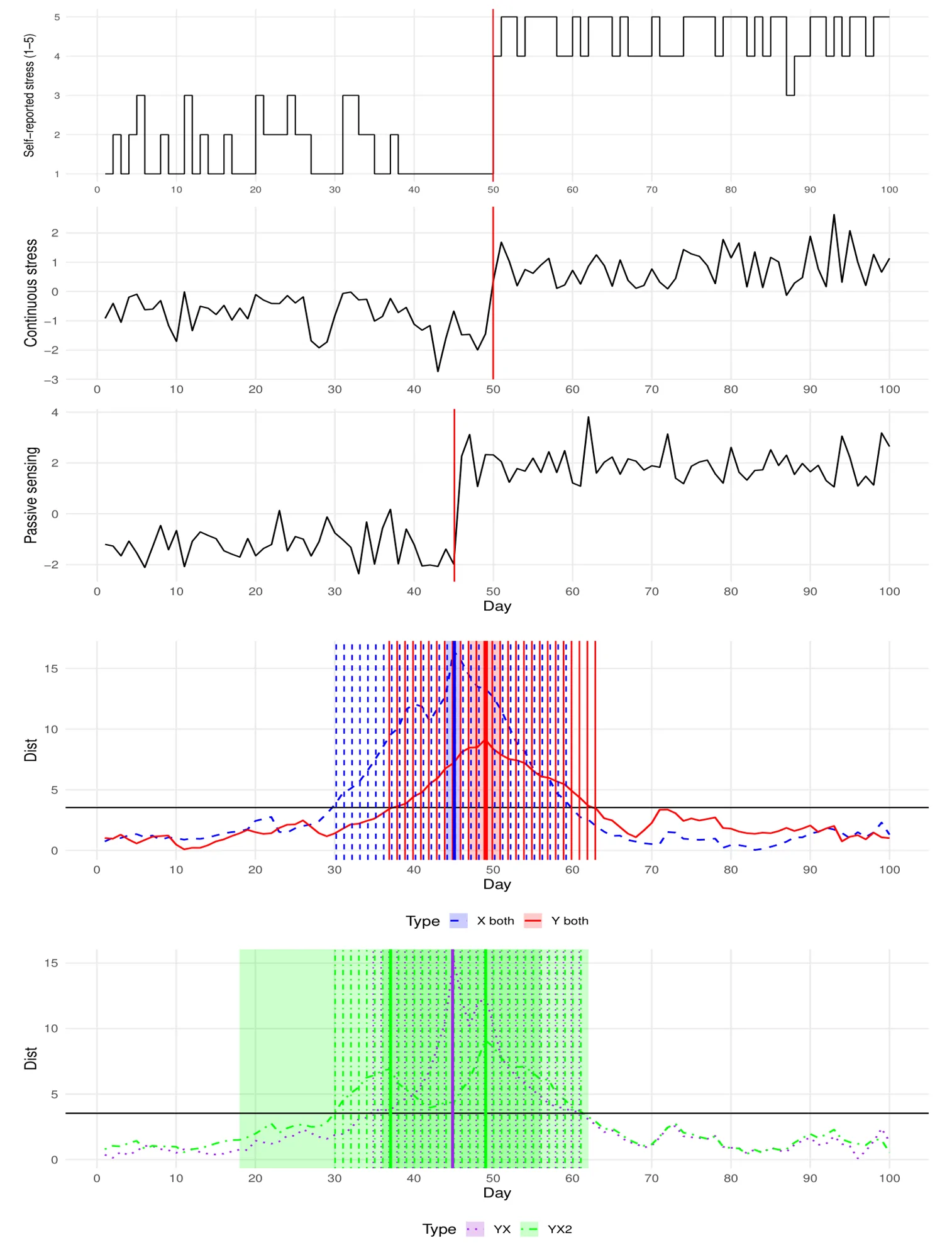
(**Top**) Simulated ordinal stress-response series, its continuous transformation $\left\{Y_{t}\right\}$, and the passive sensing series $\left\{X_{t}\right\}$. The true stress change point is at $k^{*}=50$. (**Bottom**) Joint-MOSUM distances for $\left\{Y_{t}\right\}$ and $\left\{X_{t}\right\}$ (first) and Bi-MOSUM distances for mean–mean and mean–variance changes (second). The black horizontal line is the Monte Carlo threshold; dashed vertical lines mark threshold exceedances, thick solid lines mark estimated change points, and shaded regions show pointwise confidence intervals. The resulting hotspots are [37,61] for the thresholding rule and [47,51] for the confidence-interval rule.

**Figure A2:**
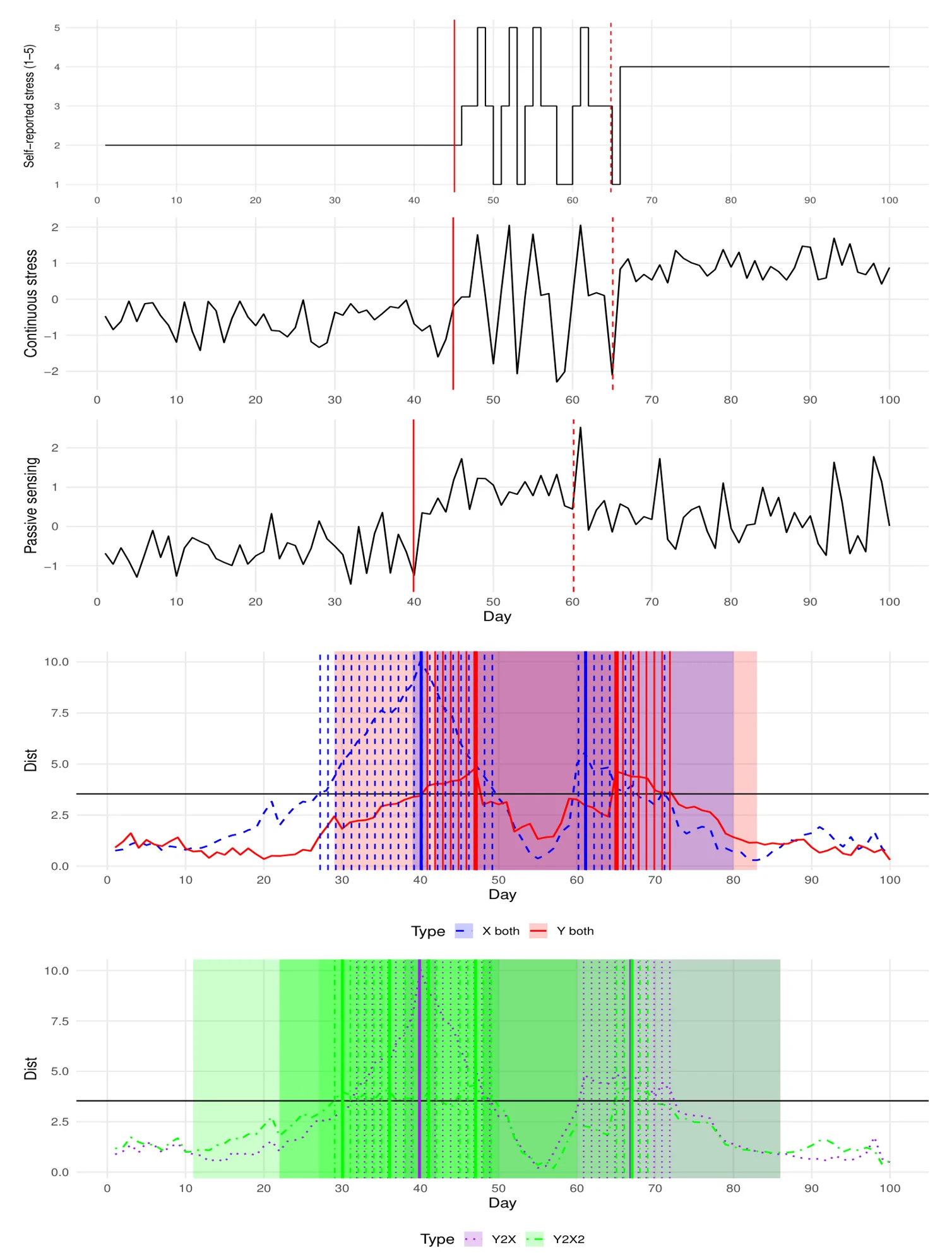
(**Top**) Simulated ordinal stress-response series, its continuous transformation $\left\{Y_{t}\right\}$, and the passive sensing series $\left\{X_{t}\right\}$. The true change points are at 45 and 65 for $\left\{Y_{t}\right\}$ and at 40 and 60 for $\left\{X_{t}\right\}$. (**Bottom**) Joint-MOSUM distances for $\left\{Y_{t}\right\}$ and $\left\{X_{t}\right\}$ (first) and Bi-MOSUM distances for variance–mean and variance–variance changes (second). The black horizontal line is the Monte Carlo threshold; dashed vertical lines mark threshold exceedances, thick solid lines mark estimated change points, and shaded regions show pointwise confidence intervals. The resulting hotspots are [40,47] and [65,72] for the thresholding rule and [29,83] for the confidence-interval rule.

In the present application, the self-reported stress series $\left\{Y_{t}\right\}$ takes values on a five-category ordinal scale. More generally, the same transformation can be applied to an ordinal stress-response series taking values in {1,…,5}. Inspired by the treatment of quantile residuals [22], we apply a smoothed and randomized inverse cumulative distribution function (CDF) transformation. Specifically, for each observation $Y_{t}=y$, y∈{1,…,5}, we define

(1) $$\begin{array}{cc} U_{t} & :={\hat{F}}_{t}\left(Y_{t}^{-}\right)+W_{t}\hat{P}\left(Y_{t}=y\right),\\ Z_{t} & :=\Phi^{-1}\left(U_{t}\right) \end{array}$$

where ${\hat{F}}_{t}\left(\cdot \right)$ denotes the estimated CDF of the observed discrete-valued series ${\left\{Y_{t}\right\}}_{t=1}^{n}$, with ${\hat{F}}_{t}\left(y^{-}\right)=\hat{P}\left(Y_{t}<y\right)$ denoting the cumulative probability immediately below category *y*. $W_{t}∼\mathrm{Unif}\left(0,1\right)$ are independent random variables drawn at each time point, and $\Phi^{-1}\left(\cdot \right)$ represents the inverse CDF of the standard normal distribution. This procedure maps each ordinal response to a continuous value within the probability interval corresponding to its observed category. Examples of this transformation are shown in the top panels of Figure A1 and Figure A2. For notational simplicity, after applying the transformation, we relabel the transformed series $\left\{Z_{t}\right\}$ as $\left\{Y_{t}\right\}$ in the subsequent methodological development.

Raw passive sensing mHealth data often exhibit a downward bias due to device non-use. The 2SpamH algorithm [23] is an unsupervised pre-processing method developed to mitigate this bias in passively collected mHealth data. The algorithm evaluates the reliability of each observation using auxiliary indicators such as screen unlock counts, battery variance, and app usage, allowing it to distinguish periods of true low activity from those resulting from device non-use. Observations identified as unreliable were treated as missing, and the missing values were imputed using the R package missForest (version 1.6.1) [24]. Further details of the 2SpamH algorithm are provided in Zhang et al. [23]. This preprocessing and imputation procedure was applied to the passive sensing data used in Section 5.4.

### 3.2. Finding Simultaneous Change Points in Univariate Series

The moving sum (MOSUM; [25,26,27]) is a widely used CPD procedure based on scan statistics [28]; by sliding the window across the series, the procedure quantifies the difference between the statistics computed from the observations before and after the window center to evaluate shifts in mean levels. Despite its simplicity, it has received less attention compared to global search methods using dynamic programming [29,30] or other rolling-window-based estimators, such as binary segmentation [31,32]. With further developments in scan-statistics-based CPD algorithms [33] and the establishment of statistical properties of the MOSUM procedure [34], it has regained attention. More recently, extensions of the MOSUM approach have been proposed to detect changes in variance [35], as well as the joint detection of mean and variance changes, referred to as Joint-MOSUM [36,37], to detect changes in both the mean and variance of individual series.

Specifically, suppose the series ${\left\{X_{t}\right\}}_{t=1}^{n}$ is given. The Joint-MOSUM algorithm identifies the time points at which changes are likely to occur. Let $\mu_{X}\left(t_{1},t_{2}\right)$ and $\sigma_{X}^{2}\left(t_{1},t_{2}\right)$ denote the population mean and variance of the observations over the interval $t_{1}$ and $t_{2}$, and their sample analogues

$$\bar{X}\left(t_{1},t_{2}\right)=\frac{1}{t_{2}-t_{1}+1}\sum_{t=t_{1}}^{t_{2}}X_{t},\;S_{X}^{2}\left(t_{1},t_{2}\right)=\frac{1}{t_{2}-t_{1}+1}\sum_{t=t_{1}}^{t_{2}}{\left(X_{t}-\bar{X}\left(t_{1},t_{2}\right)\right)}^{2}.$$

In this setting, the CPD algorithm is equivalent to performing a combined hypothesis test sequentially; for each time t=k, we test the following null hypothesis against its alternative:

(2) $$H_{0}\left(k\right):\mu_{X}\left(k-G+1,k\right)=\mu_{X}\left(k+1,k+G\right),\;\sigma_{X}^{2}\left(k-G+1,k\right)=\sigma_{X}^{2}\left(k+1,k+G\right),$$

with the corresponding alternative hypotheses $H_{A}\left(k\right)$ defined as the negation of $H_{0}\left(k\right)$, indicating that either or both of the features have changed locally at time t=k. Here, *G* denotes the half-window size, so that the local features are computed using *G* observations on each side of *k*. The choice of *G* determines the temporal scale of detection; smaller values are more sensitive to closely spaced changes, whereas larger values provide greater stability for detecting more persistent changes. In our data analysis using daily observations, we consider G=20 and 40, corresponding to local windows of 20 and 40 days on each side of a candidate change point. For notational simplicity, we suppress *G* in these features and their sample analogs hereafter. The same hypothesis test is performed for ${\left\{Y_{t}\right\}}_{t=1}^{n}$.

Following the conventional MOSUM procedure, we first consider changes in the local mean of $X_{t}$ around each candidate time point t=k. For an interior time point k=G,…,n−G, there are *G* observations available on both sides of *k*, and the local change is measured by comparing the means of these two adjacent windows. Near the beginning or end of the series, however, a complete window is not available on both sides, and boundary-adjusted statistics are used instead. Specifically,

(3) $$d\bar{X}\left(k\right)=\left\{\begin{array}{cc} C_{1}\sum_{t=1}^{k}\left(\bar{X}\left(1,2G\right)-X_{t}\right), & k=1,\ldots ,G-1,\\ \bar{X}\left(k+1,k+G\right)-\bar{X}\left(k-G+1,k\right), & k=G,\ldots ,n-G,\\ C_{n}\sum_{t=n-G+1}^{n}\left(\bar{X}\left(n-2G+1,n\right)-X_{t}\right), & k=n-G+1,\ldots ,n, \end{array}\right.$$

where $C_{1}=\frac{2}{\sqrt{k(2G-k)}}$ and $C_{n}=\frac{2}{\sqrt{\left(n+1-k)(k-n+2G\right)}}$. The first and third cases correspond to candidate change points within *G* observations of the left and right boundaries, respectively, whereas the second case corresponds to interior candidate points for which two complete windows of length *G* can be compared. The factors $C_{1}$ and $C_{n}$ are boundary normalization constants used in the MOSUM construction to adjust for the smaller and unequal effective sample sizes near the endpoints [36].

Using this local mean difference, we define the detector for a change in the mean of $X_{t}$ around t=k as

(4) $$T_{X}^{\left(1\right)}\left(k\right)=\sqrt{\frac{G}{2}}\frac{d\bar{X}\left(k\right)}{{\bar{S}}_{X}\left(k\right)},$$

where ${\bar{S}}_{X}^{2}\left(k\right)$ is the corresponding local variance estimator,

(5) $${\bar{S}}_{X}^{2}\left(k\right)=\left\{\begin{array}{cc} S_{X}^{2}\left(1,2G\right), & k=1,\ldots ,G-1,\\ \frac{1}{2}\left(S_{X}^{2}\left(k+1,k+G\right)+S_{X}^{2}\left(k-G+1,k\right)\right), & k=G,\ldots ,n-G,\\ S_{X}^{2}\left(n-2G+1,n\right), & k=n-G+1,\ldots ,n. \end{array}\right.$$

Thus, for interior points, ${\bar{S}}_{X}^{2}\left(k\right)$ averages the variability in the two windows being compared, while near the boundaries it is estimated from the corresponding 2G observations used for boundary correction. Larger values of $|T_{X}^{\left(1\right)}\left(k\right)|$ therefore indicate stronger evidence of a local mean change around *k*.

The same construction is applied to the stress-level series $\left\{Y_{t}\right\}$. In particular, the same window size *G* and the same boundary adjustments are used to define $d\bar{Y}\left(k\right)$ and ${\bar{S}}_{Y}\left(k\right)$, yielding

$$T_{Y}^{\left(1\right)}\left(k\right)=\sqrt{\frac{G}{2}}\frac{d\bar{Y}\left(k\right)}{{\bar{S}}_{Y}\left(k\right)}.$$

Using a common *G* for $X_{t}$ and $Y_{t}$ ensures that local changes in the two series are evaluated over the same temporal scale when they are subsequently combined to identify hotspots.

The studies by [35,37,38] have shown that this construction can also be applied to detect changes in variance. Specifically, the difference in the local variance of $X_{t}$ around the time point t=k, k=1,…,n, is defined as

$$dS_{X}^{2}\left(k\right)=\left\{\begin{array}{cc} C_{1}\sum_{t=1}^{k}\left(S_{X}^{2}\left(1,2G\right)-{\left(X_{t}-\bar{X}\left(1,2G\right)\right)}^{2}\right) & k=1,\ldots ,G-1,\\ S_{X}^{2}\left(k+1,k+G\right)-S_{X}^{2}\left(k-G+1,k\right) & k=G,\ldots ,n-G,\\ C_{n}\sum_{t=n-G+1}^{n}\left(S_{X}^{2}\left(n-2G+1,n\right)-{\left(X_{t}-\bar{X}\left(n-2G+1,n\right)\right)}^{2}\right) & k=n-G+1,\ldots ,n, \end{array}\right.$$

using the same constants $C_{1}$ and $C_{n}$ defined in (3). The rolling window estimator for the local change in the variance of $\left\{X_{t}\right\}$ around t=k is then defined as

(6) $$T_{X}^{\left(2\right)}\left(k\right)=\sqrt{\frac{G}{2}}\frac{dS_{X}^{2}\left(k\right)}{{\bar{V}}_{X}\left(k\right)},$$

where ${\bar{V}}_{X}^{2}\left(k\right)$ is the locally averaged estimator of the variance of the squared and centered observations at t=k, given by

(7) $${\bar{V}}_{X}^{2}\left(k\right)=\left\{\begin{array}{cc} V_{X}^{2}\left(1,2G\right), & k=1,\ldots ,G-1,\\ \frac{1}{2}\left(V_{X}^{2}\left(k+1,k+G\right)+V_{X}^{2}\left(k-G+1,k\right)\right), & k=G,\ldots ,n-G,\\ V_{X}^{2}\left(n-2G+1,n\right), & k=n-G+1,\ldots ,n, \end{array}\right.$$

where $V_{X}^{2}\left(a,b\right)=\frac{1}{b-a+1}\sum_{t=a}^{b}{\left({\left(X_{t}-\bar{X}\left(a,b\right)\right)}^{2}-S_{X}^{2}\left(a,b\right)\right)}^{2}$, using the locally averaged mean and variance defined in (5). The rolling window estimator for local changes in the variance of $\left\{Y_{t}\right\}$ around t=k, denoted by $T_{Y}^{\left(2\right)}\left(k\right)=\sqrt{\frac{G}{2}}dS_{Y}^{2}\left(k\right)/{\bar{V}}_{Y}\left(k\right)$ is defined similarly.

To test the hypothesis (2), following [37,38], we combine the mean- and variance-change detectors in (4) and (6). The joint detector at time point t=k is defined as

$$J_{X}\left(k\right)=\left(\begin{array}{c} T_{X}^{\left(1\right)}\left(k\right)\\ T_{X}^{\left(2\right)}\left(k\right) \end{array}\right)\in R^{2}.$$

The two components of $J_{X}\left(k\right)$ are generally correlated because local changes in the sample mean and variance are computed from the same observations. Under the null hypothesis, implying that there is no change point, their dependence is characterized by the population correlation matrix

$$\Gamma_{X}=\left(\begin{array}{cc} 1 & \rho_{X}\\ \rho_{X} & 1 \end{array}\right),$$

where $\rho_{X}$ is the population correlation between $X-\mu_{X}$ and ${\left(X-\mu_{X}\right)}^{2}$ which are invariant to time, given by

$$\rho_{X}:=\frac{E[{\left(X-\mu_{X}\right)}^{3}]}{\sqrt{\mathrm{Var}(X-\mu_{X})}\sqrt{\mathrm{Var}({\left(X-\mu_{X}\right)}^{2})}}=\frac{\kappa_{X}}{\sigma_{X}\nu_{X}}.$$

Its local sample analogue around t=k, denoted by ${\hat{\Gamma }}_{X}\left(k\right)$, is obtained by replacing $\rho_{X}$ with its local estimate ${\hat{\rho }}_{X}\left(k\right)$, where

(8) $${\hat{\rho }}_{X}\left(k\right)=\frac{{\bar{K}}_{X}\left(k\right)}{{\bar{S}}_{X}\left(k\right){\bar{V}}_{X}\left(k\right)},$$

with

$${\bar{K}}_{X}\left(k\right)=\frac{1}{2}\left(K_{X}\left(k+1,k+G\right)+K_{X}\left(k-G+1,k\right)\right),\;k=G,\ldots ,n-G,$$

and

$$K_{X}\left(a,b\right)=\frac{1}{b-a+1}\sum_{t=a}^{b}{\left(X_{t}-\bar{X}\left(a,b\right)\right)}^{3},$$

and define boundary versions of ${\bar{K}}_{X}\left(k\right)$ analogous to ${\bar{S}}_{X}^{2}\left(k\right)$ in (5). Thus, ${\hat{\Gamma }}_{X}\left(k\right)$ accounts for the local dependence between the mean- and variance-change detectors without requiring an explicit parametric model for that dependence.

Since the two components of $J_{X}\left(k\right)$ may be correlated, simply combining them through their squares would ignore their dependence and could overstate changes occurring along highly correlated directions. Thus, we use the Mahalanobis distance, which rescales the joint detector by its local covariance structure and provides a measure of the magnitude of the combined departure from the null hypothesis. Specifically,

(9) $$D_{X}\left(k\right)=\sqrt{J_{X}{\left(k\right)}^{\prime }{\hat{\Gamma }}_{X}{\left(k\right)}^{-1}J_{X}\left(k\right)}.$$

When the two detector components are uncorrelated, ${\hat{\Gamma }}_{X}\left(k\right)$ is approximately the identity matrix and $D_{X}\left(k\right)$ reduces to Euclidean norm of $J_{X}\left(k\right)$. In general, larger values of $D_{X}\left(k\right)$ indicate stronger evidence that the local mean, variance, or both have changed around t=k. We therefore use $D_{X}\left(k\right)$ as the test statistic at each k=1,…,n.

The Joint-MOSUM procedure identifies initial change points where the Mahalanobis distance in (9) exceeds a threshold determined by *n*, *G*, and α. Since the Joint-MOSUM statistic combines two detector components, we use a two-dimensional Monte Carlo threshold [38]. Specifically, for each replication b=1,…,B, we compute

$$D_{n}^{\left(b\right)}\left(G\right)=\max_{G+1\leq h\leq n-G}\sqrt{{\left(T_{1}^{\left(b\right)}\left(h;G\right)\right)}^{2}+{\left(T_{2}^{\left(b\right)}\left(h;G\right)\right)}^{2}},$$

where

$$T_{m}^{\left(b\right)}\left(h;G\right)=\frac{W_{m}^{\left(b\right)}\left(h+G\right)-2W_{m}^{\left(b\right)}\left(h\right)+W_{m}^{\left(b\right)}\left(h-G\right)}{\sqrt{2G}},\;m=1,2,$$

and $W_{1}^{\left(b\right)}\left(t\right)$ and $W_{2}^{\left(b\right)}\left(t\right)$ denote independent standard Gaussian random walks. We define $D_{n}\left(G,\alpha \right)$ as the empirical (1−α)-quantile of ${\left\{D_{n}^{\left(b\right)}\left(G\right)\right\}}_{b=1}^{B}$. Throughout the simulation studies and real-data application, we use window sizes G=20 and G=40, with B=1000 Monte Carlo replications. The threshold is calibrated separately for each window size. The initial change points (called initial candidates) are identified by collecting all *k* such that $D_{X}\left(k\right)>D_{n}\left(G,\alpha \right)$.

Finally, from the initial set of candidate points, we determine the finalized change points. MOSUM-type approaches are known to often detect an excessive number of change points due to the scaling (i.e., ${\bar{S}}_{X}\left(k\right)$) of the detectors [34,36]. To mitigate over-detection, the final change points are selected as the local maxima of the following expression for a small η∈(0,1):

$$\hat{k}=\underset{\bar{k}-\eta G\leq k\leq \bar{k}+\eta G}{arg max}D_{X}\left(k\right),\;$$

where $\bar{k}$ is an initial candidate satisfying the threshold criterion. Here, ηG determines the size of the neighborhood over which nearby candidate points are consolidated into a single local maximum. A smaller η uses a narrower neighborhood and therefore retains more distinct nearby peaks, increasing detection sensitivity by improving the ability to identify closely spaced changes, although potentially at the cost of more false or redundant detections. Conversely, a larger η more aggressively merges nearby candidates and thus reduces over-detection (see Section 2.2 in [36] for a related discussion). In practice, we set η=0.3 when numerous or closely spaced change points are expected, as in our simulation settings. The same procedure is applied to the other series $\left\{Y_{t}\right\}$.

### 3.3. Finding Simultaneous Change Points in Bivariate Series

Using the Joint-MOSUM approach described in Section 3.2, we extend the method to detect simultaneous changes across multiple series, referred to as Bi-MOSUM. Applying Joint- and Bi-MOSUM to $\left\{X_{t}\right\}$ and $\left\{Y_{t}\right\}$ and combining the resulting detections allows us to identify different types of combined cross-changes. It is worth noting that Joint- and Bi-MOSUM-type algorithms can be readily extended to detect other types of changes (e.g., in autocovariances), which is beyond the scope of the study.

Suppose that a pair of series ${\left\{Y_{t}\right\}}_{t=1}^{n}$ and ${\left\{X_{t}\right\}}_{t=1}^{n}$ is given. At each candidate time point t=k, we consider four possible combinations of local distributional changes across the two series. Specifically, we test

(10) $$\begin{array}{c} H_{0}^{\left(\mu_{Y},\mu_{X}\right)}\left(k\right):\mu_{Y}\left(k-G+1,k\right)=\mu_{Y}\left(k+1,k+G\right),\;\mu_{X}\left(k-G+1,k\right)=\mu_{X}\left(k+1,k+G\right),\\ H_{0}^{\left(\mu_{Y},\sigma_{X}^{2}\right)}\left(k\right):\mu_{Y}\left(k-G+1,k\right)=\mu_{Y}\left(k+1,k+G\right),\;\sigma_{X}^{2}\left(k-G+1,k\right)=\sigma_{X}^{2}\left(k+1,k+G\right),\\ H_{0}^{\left(\sigma_{Y}^{2},\mu_{X}\right)}\left(k\right):\sigma_{Y}^{2}\left(k-G+1,k\right)=\sigma_{Y}^{2}\left(k+1,k+G\right),\;\mu_{X}\left(k-G+1,k\right)=\mu_{X}\left(k+1,k+G\right),\\ H_{0}^{\left(\sigma_{Y}^{2},\sigma_{X}^{2}\right)}\left(k\right):\sigma_{Y}^{2}\left(k-G+1,k\right)=\sigma_{Y}^{2}\left(k+1,k+G\right),\;\sigma_{X}^{2}\left(k-G+1,k\right)=\sigma_{X}^{2}\left(k+1,k+G\right),\; \end{array}$$

The corresponding alternative hypothesis for each case is the negation of its null hypothesis. Thus, $H_{0}^{\left(\mu_{Y},\mu_{X}\right)}\left(k\right)$ tests whether the local means of both $Y_{t}$ and $X_{t}$ remain unchanged across the two windows adjacent to *k*, while $H_{0}^{\left(\mu_{Y},\sigma_{X}^{2}\right)}\left(k\right)$ tests for stability of the local mean of $Y_{t}$ and the local variance of $X_{t}$. Similarly, $H_{0}^{\left(\sigma_{Y}^{2},\mu_{X}\right)}\left(k\right)$ concerns the local variance of $Y_{t}$ and the local mean of $X_{t}$, and $H_{0}^{\left(\sigma_{Y}^{2},\sigma_{X}^{2}\right)}\left(k\right)$ concerns the local variances of both series. Rejection of any one of these hypotheses indicates that at least one of the corresponding features differs between the *G* observations before and after *k*, representing a local cross-change around that time point. Again, we suppress the window size *G* in the population and sample feature representations hereafter.

Specifically, consider the pair consisting of the local mean of $Y_{t}$ and the local variance of $X_{t}$ as an example. The joint detector for this combination of changes at time t=k is defined as

$$J_{YX^{2}}\left(k\right)=\left(\begin{array}{c} T_{Y}^{\left(1\right)}\left(k\right)\\ T_{X}^{\left(2\right)}\left(k\right) \end{array}\right)\in R^{2}.$$

The two components of $J_{YX^{2}}\left(k\right)$ may be correlated because they are computed from paired observations. Under the null hypothesis, their time-invariant dependence is characterized by

$$\Gamma_{YX^{2}}=\left(\begin{array}{cc} 1 & \rho_{YX^{2}}\\ \rho_{YX^{2}} & 1 \end{array}\right),$$

where

$$\rho_{YX^{2}}:=\frac{E[\left(Y-\mu_{Y}\right){\left(X-\mu_{X}\right)}^{2}]}{\sqrt{\mathrm{Var}(Y-\mu_{Y})}\sqrt{\mathrm{Var}({\left(X-\mu_{X}\right)}^{2})}}=\frac{\sigma_{YX^{2}}}{\sigma_{Y}\nu_{X}}.$$

Its local sample analog around the time point t=k, denoted by ${\hat{\Gamma }}_{YX^{2}}\left(k\right)$, is computed as

$${\hat{\rho }}_{YX^{2}}\left(k\right)=\frac{{\bar{\sigma }}_{XY^{2}}\left(k\right)}{{\bar{S}}_{Y}\left(k\right){\bar{V}}_{X}\left(k\right)},$$

where ${\bar{S}}_{Y}\left(k\right)$ and ${\bar{V}}_{X}\left(k\right)$ are locally averaged variance and variance of the squared and centered observations defined in (5) and (7), respectively, and ${\bar{\sigma }}_{YX^{2}}\left(k\right)=\frac{1}{2}\left({\hat{\sigma }}_{YX^{2}}\left(k+1,k+G\right)+{\hat{\sigma }}_{YX^{2}}\left(k-G+1,k\right)\right)$ denotes the locally averaged estimated cross-covariance at t=k, with

$${\hat{\sigma }}_{YX^{2}}\left(a,b\right)=\frac{1}{b-a+1}\sum_{t=a}^{b}\left(Y_{t}-\bar{Y}\left(a,b\right)\right){\left(X_{t}-\bar{X}\left(a,b\right)\right)}^{2}.$$

Thus, this construction accounts for the local dependence between the mean-related component of $Y_{t}$ and the variance-related component of $X_{t}$. Finally, the Mahalanobis distance used to detect the combined local change at time t=k is computed as

(11) $$D_{YX^{2}}\left(k\right)=\sqrt{J_{YX^{2}}{\left(k\right)}^{\prime }{\hat{\Gamma }}_{YX^{2}}{\left(k\right)}^{-1}J_{YX^{2}}\left(k\right)}.$$

Note that we can also consider the other three combinations of local cross-distributional shifts described in (10), where the corresponding Mahalanobis distances are denoted as $D_{YX}\left(k\right),D_{Y^{2}X}\left(k\right)$, and $D_{Y^{2}X^{2}}\left(k\right)$, k=1,2,…,n, defined analogously to (11).

Analogous to the Joint-MOSUM procedure, the Mahalanobis distance in (11) is compared with the same two-dimensional Monte Carlo threshold $D_{n}\left(G,\alpha \right)$ to identify initial candidate change points:

$$D_{YX^{2}}\left(k\right)>D_{n}\left(G,\alpha \right),\;k=1,\ldots ,n.\;$$

The same local-maxima screening rule as in the Joint-MOSUM procedure is then applied. Specifically, for each initial candidate $\bar{k}$ and a small η∈(0,1), we obtain

$$\hat{k}=\underset{\bar{k}-\eta G\leq k\leq \bar{k}+\eta G}{arg max}D_{YX^{2}}\left(k\right).\;$$

The remaining three combined hypothesis tests for local cross-changes are performed similarly.

## 4. Identifying Hotspots

We construct hotspots by combining regions identified from the stress-only and cross-series detectors. The proposed thresholding and confidence-interval rules represent potentially simultaneous or asynchronous distributional changes as temporal intervals rather than isolated change points, providing a framework for examining potential temporal concordance without assuming a specific functional relationship between the two series.

### 4.1. Thresholding Rule

We identify sets of time points for which the corresponding Mahalanobis distances exceed the given threshold; consecutive selected time points form hotspot intervals.

Continuing from the previous example, consider the detector that combines changes in the mean of $\left\{Y_{t}\right\}$ and the variance of $\left\{X_{t}\right\}$. Define the set of time points at which this cross-series detector exceeds the threshold as

(12) $${\hat{H}}_{\mathrm{Thr},YX^{2}}:=\left\{k\in \left\{1,\ldots ,n\right\}:D_{YX^{2}}\left(k\right)>D_{n}\left(G,\alpha \right)\right\}.$$

Similarly, for the Joint-MOSUM detector applied to $\left\{Y_{t}\right\}$, define

(13) $${\hat{H}}_{\mathrm{Thr},Y}:=\left\{k\in \left\{1,\ldots ,n\right\}:D_{Y}\left(k\right)>D_{n}\left(G,\alpha \right)\right\}.$$

The hotspot under the thresholding rule is then defined by the intersection of (12) and (13),

(14) $${\hat{H}}_{\mathrm{Thr}}:={\hat{H}}_{\mathrm{Thr},YX^{2}}\cap {\hat{H}}_{\mathrm{Thr},Y}.$$

Thus, a time point belongs to ${\hat{H}}_{\mathrm{Thr}}$ only when both the selected cross-series detector and the Joint-MOSUM detector for $\left\{Y_{t}\right\}$ exceed the common threshold.

Other combinations of the four cross-series Mahalanobis distances, $D_{YX}\left(k\right)$, $D_{YX^{2}}\left(k\right)$, $D_{Y^{2}X}\left(k\right)$, and $D_{Y^{2}X^{2}}\left(k\right)$, can be considered depending on the types of changes in interest. When no particular type of cross-change is specified, we consider all four cross-series detectors and define

(15) $${\hat{H}}_{\mathrm{Thr},\mathrm{full}}:=\left\{k\in \left\{1,\ldots ,n\right\}:\max\left(D_{YX}\left(k\right),D_{YX^{2}}\left(k\right),D_{Y^{2}X}\left(k\right),D_{Y^{2}X^{2}}\left(k\right)\right)>D_{n}\left(G,\alpha \right)\right\}.\;$$

In this case, the corresponding hotspot is obtained by replacing ${\hat{H}}_{\mathrm{Thr},YX^{2}}$ in (14) with ${\hat{H}}_{\mathrm{Thr},\mathrm{full}}$ defined in (15), yielding

(16) $${\hat{H}}_{\mathrm{Thr}}={\hat{H}}_{\mathrm{Thr},\mathrm{full}}\cap {\hat{H}}_{\mathrm{Thr},Y}.$$

More generally, ${\hat{H}}_{\mathrm{Thr},YX^{2}}$ in (12) may be replaced by a set defined using any subset of the four cross-series detectors, depending on the distributional changes under consideration. We refer to this construction as the thresholding rule.

### 4.2. Confidence Interval Rule

CPD algorithms are designed to detect abrupt shifts rather than gradual transitions. Because our goal is to construct hotspot intervals rather than report isolated change point estimates, we augment the estimated change points with intervals that quantify uncertainty around their locations. While extensive research has focused on developing CPD algorithms, relatively few studies have addressed uncertainty quantification, aside from a few Bayesian approaches [39,40]. In frequentist approaches, methods based on controlling the family-wise error rate [41], false discovery rate [42], and post-selection inference [43] have been proposed. However, these approaches are not directly designed to produce the interval-valued representation of change point uncertainty required for our hotspot construction, as illustrated in Figure 1.

Recently, Cho and Kirch [44] proposed two methods for constructing confidence intervals for change points in univariate MOSUM: one based on pointwise coverage and the other on controlling the family-wise error rate. As our primary objective is to construct intervals around individual estimated change points and examine their overlap across detectors, we adopt the pointwise method in our framework.

Confidence intervals for change points from the Joint- and Bi-MOSUM procedures can be constructed analogously to those for univariate MOSUM change points, following the bootstrap approach in Section 2.3 of [44] and Section 2.7 of [36]. For the Bi-MOSUM procedure, the paired observations $\left(Y_{t},X_{t}\right)$ are resampled jointly within each estimated segment.

Continuing from the previous example, suppose there are ${\hat{K}}_{YX^{2}}=\left|{\hat{T}}_{YX^{2}}\right|$ estimated change points associated with the detector combining the mean of $Y_{t}$ and the variance of $X_{t}$. We order them as

$$0={\hat{k}}_{YX^{2},0}<{\hat{k}}_{YX^{2},1}<\ldots <{\hat{k}}_{YX^{2},{\hat{K}}_{YX^{2}}}<{\hat{k}}_{YX^{2},{\hat{K}}_{YX^{2}}+1}=n.$$

Define the corresponding segments as

$$I_{YX^{2},j}=\left\{{\hat{k}}_{YX^{2},j-1}+1,\ldots ,{\hat{k}}_{YX^{2},j}\right\},\;j=1,\ldots ,{\hat{K}}_{YX^{2}}+1.$$

Within each segment, we draw a random sample of size $|I_{YX^{2},j}|$ with replacement from the paired observations $\left\{\left(Y_{t},X_{t}\right):t\in I_{YX^{2},j}\right\}$. Resampling the two series jointly preserves the contemporaneous dependence between $Y_{t}$ and $X_{t}$ within each estimated segment. Repeating this procedure across all segments yields bootstrap samples

$$\left\{\left(Y_{1}^{\left(b\right)},X_{1}^{\left(b\right)}\right),\ldots ,\left(Y_{n}^{\left(b\right)},X_{n}^{\left(b\right)}\right)\right\},\;b=1,\ldots ,B.$$

For each bootstrap replication, the $j^{\mathrm{th}}$ change point is re-estimated locally as

$${\hat{k}}_{YX^{2},j}^{\left(b\right)}=\underset{{\hat{k}}_{YX^{2},j}-G\leq k\leq {\hat{k}}_{YX^{2},j}+G}{arg max}D_{YX^{2}}^{\left(b\right)}\left(k\right).$$

For each original estimate ${\hat{k}}_{YX^{2},j}$, let $M_{YX^{2},j}\left(\alpha \right)$ denote the 100(1−α)th percentile of

$$\left\{|{\hat{k}}_{YX^{2},j}^{\left(1\right)}-{\hat{k}}_{YX^{2},j}\left|,\ldots ,\right|{\hat{k}}_{YX^{2},j}^{\left(B\right)}-{\hat{k}}_{YX^{2},j}|\right\}.$$

The resulting pointwise 100(1−α)% confidence interval is

$${\mathrm{CI}}_{YX^{2},j}\left(\alpha \right)=\left[\max\left(1,{\hat{k}}_{YX^{2},j}-M_{YX^{2},j}\left(\alpha \right)\right),\min\left({\hat{k}}_{YX^{2},j}+M_{YX^{2},j}\left(\alpha \right),n\right)\right],\;j=1,\ldots ,{\hat{K}}_{YX^{2}}.$$

Define the union of these confidence intervals as

(17) $${\hat{H}}_{\mathrm{CI},YX^{2}}\left(\alpha \right):=\overset{{\hat{K}}_{YX^{2}}}{\underset{j=1}{⋃}}{\mathrm{CI}}_{YX^{2},j}\left(\alpha \right).$$

Similarly, let ${\hat{K}}_{Y}=\left|{\hat{T}}_{Y}\right|$ and define

(18) $${\hat{H}}_{\mathrm{CI},Y}\left(\alpha \right):=\overset{{\hat{K}}_{Y}}{\underset{j=1}{⋃}}{\mathrm{CI}}_{Y,j}\left(\alpha \right)$$

denote the union of confidence intervals corresponding to the Joint-MOSUM change points detected in $\left\{Y_{t}\right\}$. The hotspot under the confidence interval rule is then defined as an intersection of (17) and (18):

(19) $${\hat{H}}_{\mathrm{CI}}\left(\alpha \right):={\hat{H}}_{\mathrm{CI},YX^{2}}\left(\alpha \right)\cap {\hat{H}}_{\mathrm{CI},Y}\left(\alpha \right).$$

Thus, the CI-based hotspot consists of time points lying within confidence intervals associated with both the selected cross-series changes and changes detected in $\left\{Y_{t}\right\}$.

If all four types of cross-series changes are considered, define

(20) $$\begin{array}{cc} \; & {\hat{H}}_{\mathrm{CI},\mathrm{full}}\left(\alpha \right)\\ := & \left(\overset{{\hat{K}}_{YX}}{\underset{j_{1}=1}{⋃}}{\mathrm{CI}}_{YX,j_{1}}\left(\alpha \right)\right)\cup \left(\overset{{\hat{K}}_{YX^{2}}}{\underset{j_{2}=1}{⋃}}{\mathrm{CI}}_{YX^{2},j_{2}}\left(\alpha \right)\right)\cup \left(\overset{{\hat{K}}_{Y^{2}X}}{\underset{j_{3}=1}{⋃}}{\mathrm{CI}}_{Y^{2}X,j_{3}}\left(\alpha \right)\right)\cup \left(\overset{{\hat{K}}_{Y^{2}X^{2}}}{\underset{j_{4}=1}{⋃}}{\mathrm{CI}}_{Y^{2}X^{2},j_{4}}\left(\alpha \right)\right). \end{array}$$

The corresponding hotspot is obtained by replacing ${\hat{H}}_{\mathrm{CI},YX^{2}}\left(\alpha \right)$ in (19) with ${\hat{H}}_{\mathrm{CI},\mathrm{full}}\left(\alpha \right)$ in (20):

(21) $${\hat{H}}_{\mathrm{CI}}\left(\alpha \right)={\hat{H}}_{\mathrm{CI},\mathrm{full}}\left(\alpha \right)\cap {\hat{H}}_{\mathrm{CI},Y}\left(\alpha \right).$$

More generally, ${\hat{H}}_{\mathrm{CI},YX^{2}}\left(\alpha \right)$ in (19) may be replaced by the union of confidence intervals associated with any selected subset of the four cross-series detector combinations, depending on the distributional changes under consideration. We refer to this construction as the confidence interval rule. For both hotspot rules, the bivariate detectors may respond to changes in either series, as their alternative hypotheses do not require changes in both series. Accordingly, the resulting hotspots are interpreted as candidate regions for examining temporal concordance between the two series.

### 4.3. Illustration of Algorithm

Two illustrative examples of hotspot identification are shown in Figure A1 and Figure A2, provided in Appendix A.1.

The first example in Figure A1 shows a scenario in which the simulated ordinal stress-response series $\left\{Y_{t}\right\}$ undergoes a single change in its mean, while the corresponding passive sensing variable exhibits simultaneous changes in both its mean and variance, with a five–time-point lag between the two series. The second example in Figure A2 demonstrates a scenario with two variance changes in the simulated ordinal stress-response series and corresponding changes in the passive sensing series, again with a five-time-point lag between the two series.

## 5. Numerical Studies

The numerical studies consist of three parts. First, we examine whether jointly detecting multiple types of distributional changes improves change point detection when different types of changes coexist. We also compare the proposed Joint- and Bi-MOSUM approaches with benchmark CPD methods, focusing on their ability to identify change points sufficiently close to the true locations for subsequent hotspot construction.

Second, we investigate the properties of the proposed thresholding and confidence-interval hotspot rules, focusing on their ability to retain true change points while producing relatively concise intervals, particularly when changes in the two series occur asynchronously. We further examine their behavior under no-change and single-series-change scenarios, as well as their sensitivity to temporal dependence and randomized Likert transformations.

Finally, we apply the proposed procedures to real-world data to illustrate their practical application, including comparisons with stress-only change point detection and an assessment of sensitivity to temporal shifts.

### 5.1. Simulation on Change Points

We begin by evaluating change point detection in a univariate time series. Throughout the simulation studies, the sample size is fixed at n=100, reflecting the relatively short time series considered in the motivating application. We consider a no-change scenario as well as scenarios with one, two, or three true change points. Under the no-change scenario, the observations are independently generated from N(0,1). For the change point scenarios, the true change points are located at $k^{*}=50$ for the one-change scenario, $k^{*}$= 40, 60 for the two-change scenario, and $k^{*}$ = 25, 50, 75 for the three-change scenario.

For the scenarios containing change points, within each segment *j*, observations are independently generated from $X_{t}∼N\left(\mu_{j},\sigma_{j}^{2}\right)$, where the segment-specific mean and standard deviation are independently generated as $\mu_{j}∼\mathrm{Unif}\left(-a,a\right)$ and $\sigma_{j}∼\mathrm{Unif}\left(\sigma_{\min},\sigma_{\max}\right)$. We consider six settings that vary the range of the segment means and standard deviations:

$$\begin{array}{cc} \mathrm{Case}\;1: & \;a=2,\sigma_{\min}=0.1,\sigma_{\max}=0.4,\\ \mathrm{Case}\;2: & \;a=2,\sigma_{\min}=0.1,\sigma_{\max}=0.8,\\ \mathrm{Case}\;3: & \;a=2,\sigma_{\min}=0.4,\sigma_{\max}=0.8,\\ \mathrm{Case}\;4: & \;a=1,\sigma_{\min}=0.1,\sigma_{\max}=0.4,\\ \mathrm{Case}\;5: & \;a=1,\sigma_{\min}=0.1,\sigma_{\max}=0.8,\\ \mathrm{Case}\;6: & \;a=1,\sigma_{\min}=0.4,\sigma_{\max}=0.8. \end{array}$$

Thus, the six settings span different combinations of the magnitude of between-segment mean variation and within-segment variability. Each setting is replicated 500 times.

We apply the Joint-MOSUM procedure described in Section 3 using window sizes G=20 and G=40. We compare it with several established univariate change point detection methods representing different detection strategies. PELT [30], implemented using the R package changepoint (version 2.3) [45], is applied to changes in both the mean and variance using the MBIC penalty and a Gaussian likelihood. Wild binary segmentation (WBS) [32], implemented in the R package breakfast (version 2.5), searches over randomly selected subintervals and is designed to identify multiple, potentially closely spaced changes. We also include stepR (version 2.1-11) [46] and the conventional univariate MOSUM procedure of Eichinger and Kirch [34], implemented in the R package mosum (version 1.2.7), with window sizes G=20 and G=40.

Our primary evaluation criterion is not exact localization of every change point, but whether the estimated change points identify the neighborhoods of the true change locations that can subsequently serve as anchors for hotspot construction. Because hotspots are defined as temporal intervals, small localization errors are less consequential than failing to identify the neighborhood of a true distributional change. We therefore evaluate change point detection within a tolerance of τ time points.

For each replication i=1,…,500, let $T^{*}=\left\{t_{1}^{*},\ldots ,t_{K^{*}}^{*}\right\}$ denote the set of true change points and ${\hat{T}}^{\left(i\right)}=\left\{{\hat{t}}_{1}^{\left(i\right)},\ldots ,{\hat{t}}_{{\hat{K}}^{\left(i\right)}}^{\left(i\right)}\right\}$ denote the set of estimated change points. We define the detection rate (DR) as

$$\mathrm{DR}\left(\tau \right)=\frac{1}{500}\sum_{i=1}^{500}1_{\left\{\max_{1\leq \ell \leq K^{*}}\min_{1\leq j\leq {\hat{K}}^{\left(i\right)}}\left|{\hat{t}}_{j}^{\left(i\right)}-t_{\ell }^{*}\right|\leq \tau \right\}},$$

Thus, DR is the proportion of Monte Carlo replications in which every true change point is accompanied by at least one estimated change point within the specified tolerance. Multiple estimated change points within the same tolerance neighborhood do not increase the detection rate, as successful detection is determined by whether the neighborhood of each true change point is identified. Unlike conventional statistical power, DR reflects the probability of recovering all true change point neighborhoods within a replication, providing a direct measure of complete change point recovery within the specified tolerance.

To quantify the failure to match any true change point, we define the no-match rate (NMR) as

$$\mathrm{NMR}\left(\tau \right)=\frac{\sum_{i=1}^{500}1_{\left\{{\hat{K}}^{\left(i\right)}\geq 1,\;\min_{1\leq j\leq {\hat{K}}^{\left(i\right)}}\min_{1\leq \ell \leq K^{*}}\left|{\hat{t}}_{j}^{\left(i\right)}-t_{\ell }^{*}\right|>\tau \right\}}}{\sum_{i=1}^{500}1_{\left\{{\hat{K}}^{\left(i\right)}\geq 1\right\}}}.$$

NMR measures the proportion of replications with at least one estimated change point in which none of the estimates matches any true change point within the specified tolerance. Replications with no detected change points are excluded from the denominator. Unlike the conventional false discovery rate, NMR evaluates complete failure to match any true change point rather than the proportion of individual false discoveries. For the univariate simulations, we set τ=5 for both DR and NMR. For the no-change scenario, where $K^{*}=0$, neither DR nor NMR is applicable. Instead, we report the false positive rate (FPR), defined as the proportion of replications in which at least one change point is detected:

$$\mathrm{FPR}=\frac{1}{500}\sum_{i=1}^{500}1_{\left\{{\hat{K}}^{\left(i\right)}>0\right\}}.\;$$

Table 1 presents the change point detection results for the univariate simulations. For a single change point, Joint-MOSUM with G=40 achieves the highest DR across all six cases, with DR ranging from 0.642 to 0.940. When two change points are present, the smaller window becomes more effective: Joint-MOSUM with G=20 attains the highest DR in four of the six cases and ties with WBS in Case 3, while WBS performs best in Case 1. This pattern becomes more pronounced with three change points, where Joint-MOSUM with G=20 achieves the highest DR in all six cases. In contrast, the performance of G=40 deteriorates substantially as the number of change points increases, reflecting the difficulty of using a relatively large window when multiple changes occur within a short series. The NMR values are generally low across methods, particularly in the multiple-change settings, although no single method uniformly achieves the lowest NMR across all cases. These values reflect the frequency with which all estimated change points fail to match any true change point, rather than the proportion of individual false detections. Overall, these results illustrate the role of window size in the proposed procedure. A larger window is more effective when fewer changes are present, whereas a smaller window performs better when multiple changes occur within a short series.

As an additional assessment, Table A1 in Appendix A.2 reports FPRs under the no-change scenario. The benchmark methods generally show lower FPRs, while Joint-MOSUM yields FPRs of 0.172 for G=20 and 0.112 for G=40. Thus, the higher DR achieved by Joint-MOSUM in several change point scenarios should be considered alongside its greater frequency of false positive detections under the no-change scenario.

For the bivariate simulation, we evaluate the Bi-MOSUM procedure and compare it with benchmark methods for multivariate change point detection. As in the univariate simulation, each of the two series has length n=100 and is generated independently according to the same segment-wise Gaussian model and the six parameter settings described above. The segment-specific means and standard deviations are generated separately for the two series. We consider three configurations of change locations: (i) one change at $k^{*}=60$ in the first series and one change at $k^{*}=40$ in the second series; (ii) one change at $k^{*}=50$ in the first series and two changes at $k^{*}=40,60$ in the second series; and (iii) two changes at $k^{*}=40,60$ in the first series and two changes at $k^{*}=30,70$ in the second series. Each simulation setting is replicated 500 times.

For Bi-MOSUM, we apply all four combinations of distributional changes, mean–mean, mean–variance, variance–mean, and variance–variance, because the types of changes are not assumed to be known in advance. The estimated change points obtained from the four detectors are then combined into a single set of candidate change locations. We use window sizes G=20 and G=40. We compare Bi-MOSUM with several established multivariate change point detection methods. Specifically, we consider the nonparametric method (ECP) of Matteson and James [20], implemented in the R package ecp [47] (version 3.1.3); sparsified binary segmentation (SBS) [48]; and double CUSUM binary segmentation (DCBS) [49], with SBS and DCBS implemented in the R package hdbinseg (version 1.0.3). We also include the nonparametric multivariate change point method (NMP) of Padilla et al. [50], implemented using the publicly available authors’ code (available at https://github.com/HaotianXu/changepoints/tree/main (accessed on 22 October 2026)).

For the bivariate simulations, the true change locations from the two series are pooled into a single set of distinct change points. Thus, the three configurations contain $K^{*}=2$, $K^{*}=3$, and $K^{*}=4$ distinct true change locations, respectively. We set τ=5 for DR and τ=0 for NMR, so that DR allows for small localization errors while NMR evaluates the absence of any exact match between estimated and true change points.

Table 2 presents the change point detection results for the bivariate simulations. When each series contains a single change point, Bi-MOSUM with G=40 performs best across all six cases, achieving the highest DR and the lowest NMR throughout. When the number of changes increases, however, the smaller window becomes more effective. For the (1,2) and (2,2) configurations, Bi-MOSUM with G=20 attains the highest DR in all six cases and generally maintains lower NMR values than the benchmark methods. In contrast, the DR of Bi-MOSUM with G=40 decreases substantially in the (2,2) configuration, even though its NMR remains relatively low. This difference reflects the distinct evaluation criteria: DR requires recovery of all true change point neighborhoods, whereas NMR only assesses whether at least one estimated change point matches a true change point exactly. Overall, the bivariate results show a pattern consistent with the univariate simulations: a larger window is advantageous when fewer changes are present, whereas a smaller window is better suited to settings with multiple changes within a short series.

### 5.2. Simulation on Hotspots

We evaluate the hotspot definitions based on their ability to retain the relevant change locations while producing relatively concise temporal intervals, particularly when changes in the two series occur asynchronously.

We consider two hotspot constructions: the thresholding rule (Thrs) and the confidence-interval rule (CI). Since the types of distributional changes are not assumed to be known in advance, we include all four cross-series detector combinations. Accordingly, the thresholding hotspot is defined by (16), using ${\hat{H}}_{\mathrm{Thr},\mathrm{full}}$ in (15), and the CI-based hotspot is defined by (21), using ${\hat{H}}_{\mathrm{CI},\mathrm{full}}\left(\alpha \right)$ in (20). We set α=0.05 for both rules.

The sample size is fixed at n=100, and we consider the same six signal-strength cases described in Section 5.1. Each series is generated independently according to the segment-wise Gaussian model used in Section 5.1, with the segment-specific means and standard deviations generated separately for $\left\{X_{t}\right\}$ and $\left\{Y_{t}\right\}$. We consider scenarios containing either one or two change points. For $\left\{X_{t}\right\}$, the change point is located at $k^{*}=50$ in the one-change scenario, whereas the two change points are located at $k^{*}=40$ and 60. For $\left\{Y_{t}\right\}$, the corresponding change points are shifted by δ, occurring at $k^{*}=50+\delta$ in the one-change scenario and at $k^{*}=40+\delta$ and 60+δ in the two-change scenario, where δ∈{0,5}. Thus, δ=0 represents synchronous changes across the two series, whereas δ=5 represents a temporal displacement of five time points. Each setting is replicated 200 times. For each replication, we apply the Joint- and Bi-MOSUM procedures using window sizes G=20 and G=40. For the thresholding rule, the Monte Carlo threshold is calibrated using the same window size *G* as that used in the corresponding MOSUM procedure. For the CI rule, we use B=1000 bootstrap replications.

We evaluate hotspot performance using two measures: the hit rate and the hotspot interval length. The hit rate is defined as the proportion of true change points in $\left\{Y_{t}\right\}$ contained in the estimated hotspots. For replication *i*, let ${\hat{H}}^{\left(i\right)}$ denote the estimated hotspot set and let $T_{Y}^{*}=\left\{t_{1}^{*},\ldots ,t_{K^{*}}^{*}\right\}$ denote the true change points in $\left\{Y_{t}\right\}$. The hit rate is defined as

$$\mathrm{Hit}\mathrm{rate}=\frac{1}{200K^{*}}\sum_{i=1}^{200}\sum_{\ell =1}^{K^{*}}1_{\left\{t_{\ell }^{*}\in {\hat{H}}^{\left(i\right)}\right\}}.$$

Thus, the hit rate measures the average proportion of true change points retained by the estimated hotspots across the 200 replications. The hotspot interval length is defined as the length of the connected hotspot interval associated with each true change point. If a true change point is contained in $\hat{H}$, we record the length of the connected hotspot interval containing that change point. If the true change point is not contained in any estimated hotspot, we assign a length of n=100, representing the worst case in which the entire observation period would need to be considered. These lengths are then averaged across true change points and simulation replications. Taken together, the hit rate measures whether the relevant change locations are retained, whereas the interval length measures their temporal localization.

Table 3 presents the simulation results. For the one-change scenarios, both hotspot rules generally achieve high hit rates, with the CI rule producing shorter average hotspot lengths. The thresholding rule with G=40 consistently achieves higher hit rates than with G=20, although its hotspots tend to cover broader temporal regions. When two change points are present, the CI rule with G=40 exhibits substantially lower hit rates and larger average hotspot lengths, particularly under temporal displacement (δ=5), whereas the thresholding rule remains relatively stable. Across both rules, hit rates generally decrease as the signal becomes weaker. Since a length of n=100 is assigned when a true change point is missed, the average hotspot length reflects both temporal localization and missed detections. Overall, these results highlight the trade-off between detection and localization across hotspot rules and window sizes.

To further evaluate the behavior of the hotspot procedures, we consider three scenarios: (i) neither series contains a change point, (ii) only $\left\{X_{t}\right\}$ contains a change point, and (iii) only $\left\{Y_{t}\right\}$ contains a change point. In the no-change setting, both series are generated independently as standard Gaussian sequences. In the one-series-change settings, the changing series contains a single change point at t=50 and is generated according to the segment-wise Gaussian model described above using Cases 1, 2, and 4, whereas the other series is generated as an independent standard Gaussian sequence with no change point. Each setting is replicated 200 times, and both the thresholding and confidence-interval hotspot rules are applied with G=20 and G=40 and α=0.05. Since the proposed hotspot construction is anchored to distributional changes in $\left\{Y_{t}\right\}$, we distinguish the evaluation of the first two scenarios from that of the third. When neither series changes or only $\left\{X_{t}\right\}$ changes, any identified hotspot is regarded as spurious. In contrast, when only $\left\{Y_{t}\right\}$ changes, the hotspot rules in (16) and (21) may still identify nonempty regions, as the bivariate detectors can respond to distributional changes in $\left\{Y_{t}\right\}$ even when $\left\{X_{t}\right\}$ remains stationary. Therefore, we evaluate the absence of hotspot detection rather than treating nonempty estimated hotspots as false detections in this scenario.

For the no-change and *X*-only change scenarios, we quantify spurious hotspot detection using the false hotspot rate (FHR) and average false hotspot length (FHL), defined as

$$\mathrm{FHR}=\frac{1}{200}\sum_{i=1}^{200}1_{\left\{{\hat{H}}^{\left(i\right)}\neq \emptyset \right\}},\;\mathrm{FHL}=\frac{1}{200}\sum_{i=1}^{200}\left|{\hat{H}}^{\left(i\right)}\right|,$$

where ${\hat{H}}^{\left(i\right)}$ denotes the estimated hotspot set in replication *i*, and $|{\hat{H}}^{\left(i\right)}|$ denotes its cardinality, representing the total number of time points covered by the estimated hotspots, with a value of zero when no hotspot is detected. Lower FHR and FHL indicate better control of spurious hotspot identification. For the *Y*-only change scenario, we instead consider the no-hotspot rate (NHR) and mean hotspot length (MHL), defined as

$$\mathrm{NHR}=\frac{1}{200}\sum_{i=1}^{200}1_{\left\{{\hat{H}}^{\left(i\right)}=\emptyset \right\}},\;\mathrm{MHL}=\frac{1}{200}\sum_{i=1}^{200}\left|{\hat{H}}^{\left(i\right)}\right|.$$

NHR measures the proportion of replications in which no hotspot is identified despite a distributional change in $\left\{Y_{t}\right\}$, whereas MHL summarizes the average extent of the identified hotspot regions. A lower NHR indicates fewer empty hotspot sets when $\left\{Y_{t}\right\}$ changes.

Table A2 in Appendix A.2 summarizes hotspot detection under the three scenarios. When neither series changes, the FHR ranges from 0.135 to 0.160, while the *X*-only change scenario yields FHR values between 0.150 and 0.225. Although the FHR values are generally comparable between the two hotspot rules, the CI rule produces substantially larger FHL values than the thresholding rule, indicating greater temporal coverage of spurious hotspots on average. In contrast, when only $\left\{Y_{t}\right\}$ changes, the NHR ranges from 0.015 to 0.150, with consistently lower values for G=40 than for G=20. These results indicate that both rules frequently produce nonempty hotspots when $\left\{Y_{t}\right\}$ changes, although NHR does not evaluate whether the true change point is contained within the identified regions.

### 5.3. Sensitivity Analyses

We conduct two additional sensitivity analyses to examine the sensitivity of hotspot identification. First, we introduce serial dependence to assess sensitivity to temporal autocorrelation. Second, we examine the variability of identified hotspots across repeated randomized transformations of ordinal Likert responses. Unless otherwise specified, the hotspot procedures and tuning parameters are the same as those used in Section 5.2.

#### 5.3.1. Sensitivity to Temporal Dependence

The simulations above generate observations independently within each segment. To examine sensitivity to temporal dependence, we replace the independent Gaussian errors with stationary AR(1) processes. Specifically, for each series, we generate

$$E_{t}=\rho E_{t-1}+\sqrt{1-\rho^{2}}\epsilon_{t},\;\epsilon_{t}\overset{i.i.d.}{∼}N\left(0,1\right),$$

with $E_{1}∼N\left(0,1\right)$, so that the marginal variance of $E_{t}$ is one. The observed series are then generated within segment *j* as

$$X_{t}=\mu_{X,j}+\sigma_{X,j}E_{X,t},\;Y_{t}=\mu_{Y,j}+\sigma_{Y,j}E_{Y,t},$$

where the segment-specific means and standard deviations follow the same data-generating settings as in Section 5.1. We consider ρ=0.3 and ρ=0.6 and focus on the one-change point setting under Cases 1, 2, and 4. The change point in $\left\{X_{t}\right\}$ is located at t=50, while that in $\left\{Y_{t}\right\}$ is located at t=50+δ, with δ∈{0,5}. Each setting is replicated 200 times. We apply both hotspot rules with G=20 and G=40, retaining the original threshold calibration and bootstrap procedures without additional adjustment for temporal dependence. Performance is evaluated using the hit rate and hotspot interval length defined in Section 5.2.

Table A3 in Appendix A.2 presents the results under temporal dependence. Across the considered settings, both hotspot rules maintain relatively high hit rates, ranging from 0.820 to 0.980. For the thresholding rule, G=40 consistently achieves higher hit rates than G=20, although the resulting hotspots cover longer temporal intervals. The CI rule generally produces shorter intervals while maintaining relatively high hit rates. Increasing ρ from 0.3 to 0.6 does not lead to a systematic decline in hit rates, although the CI-based hotspot lengths increase across all considered settings. Overall, these results suggest that the procedures retain relatively high hit rates under the examined levels of temporal dependence, while temporal localization, particularly under the CI rule, is sensitive to stronger autocorrelation.

#### 5.3.2. Sensitivity to the Randomized Likert Transformation

We next examine the sensitivity of the hotspot results to the random draws used in the transformation of ordinal Likert responses. To generate a five-level ordinal series with a known underlying change structure, we first generate a latent continuous process $\left\{Y_{t}^{*}\right\}$ according to the segment-wise Gaussian model used in the main simulations. The latent observations are then discretized into five ordinal categories,

$$Y_{t}=r\;\mathrm{if}\;c_{r-1}<Y_{t}^{*}\leq c_{r},\;r=1,\ldots ,5,\;$$

where $c_{0}=-\infty$, $c_{5}=\infty$, and $c_{r}=\Phi^{-1}\left(r/5\right)$ for r=1,…,4. The resulting series $\left\{Y_{t}\right\}$ therefore takes values in {1,…,5}, analogous to the ordinal stress responses considered in the data application. For each simulated dataset, we hold the ordinal series $\left\{Y_{t}\right\}$ and the corresponding continuous series $\left\{X_{t}\right\}$ fixed and apply the randomized transformation in (1) M=10 times. For the $m^{\mathrm{th}}$ transformation, m=1,…,M, a new independent sequence ${\left\{W_{t}^{\left(m\right)}\right\}}_{t=1}^{n}$ is generated from Unif(0,1) and substituted for ${\left\{W_{t}\right\}}_{t=1}^{n}$ in (1), yielding a transformed series ${\left\{Z_{t}^{\left(m\right)}\right\}}_{t=1}^{n}$. The hotspot procedure is then applied separately to each transformed version while all other components of the simulated dataset are held unchanged. This design isolates variation in the estimated hotspots attributable solely to the random draws in the transformation.

We consider Cases 1, 2, and 4 in the one change point setting with δ∈{0,5} and generate 200 independent datasets for each setting. Both hotspot rules are evaluated with G=20 and G=40. For each dataset, the randomized transformation is repeated M=10 times while the underlying ordinal series and the corresponding $\left\{X_{t}\right\}$ series are held fixed. To assess the stability of the estimated hotspot regions with respect to the random draws, we compute the average pairwise Jaccard similarity among the *M* estimated hotspot sets. For replication *i*, this is defined as

$$J^{\left(i\right)}=\frac{2}{M(M-1)}\sum_{1\leq m<m^{\prime }\leq M}\frac{\left|{\hat{H}}^{\left(i,m\right)}\cap {\hat{H}}^{\left(i,m^{\prime }\right)}\right|}{\left|{\hat{H}}^{\left(i,m\right)}\cup {\hat{H}}^{\left(i,m^{\prime }\right)}\right|}.\;$$

Here, ${\hat{H}}^{\left(i,m\right)}$ denotes the hotspot set obtained from the $m^{\mathrm{th}}$ randomized transformation of dataset *i*. When both hotspot sets in a pair are empty, their Jaccard similarity is defined as one. We report the average of $J^{\left(i\right)}$ across the simulated datasets, with values closer to one indicating greater stability of the estimated hotspot regions with respect to the random draws used in the transformation.

Table A4 in Appendix A.2 summarizes the stability of hotspot identification under repeated randomized Likert transformations. Across Cases 1, 2, and 4 and both settings δ∈{0,5}, the average pairwise Jaccard similarity ranged from 0.676 to 0.784 for the thresholding rule and from 0.623 to 0.668 for the CI rule. The thresholding rule consistently produced higher similarity than the CI rule across all simulation settings. Moreover, using G=40 resulted in higher similarity than G=20 for both rules, suggesting greater stability under repeated random transformations. Overall, these results indicate that the identified hotspot regions exhibit moderate to relatively high agreement across randomized transformations, although some variability remains due to the randomization.

### 5.4. Data Application

We apply the hotspot rules examined in Section 5.2 to data from the ALACRITY Phase I study, conducted by the Weill Cornell ALACRITY Center (P50MH113838). The ALACRITY Phase I program comprised three intervention studies, REDS, RELIEF, and PROTECT, that targeted different clinical and community populations. REDS was a community-care intervention for senior center clients, RELIEF was a psychotherapy for primary-care patients with chronic pain and depressive symptoms, and PROTECT was a psychotherapy for elder-abuse victims with depression [51]. The analytic data available for the present study included 104 participants across these three studies, including 35 participants from RELIEF. Among the 35 RELIEF participants, four participants (sternrelief5, sternrelief6, sternrelief15, and sternrelief16) were selected for the present methodological illustration based on the availability of longitudinal self-report and passive sensing data and their representation of diverse temporal patterns across the measured variables. Participant-specific diagnostic information was not available in the analytic data. Instead, we report the available baseline clinical measures and study-level treatment context rather than assigning individual diagnoses. Participant-level demographic, clinical, and observational characteristics are summarized in Table A5. All four participants were female and ranged in age from 60.2 to 82.5 years (mean = 72.4 years). Depressive symptom severity was assessed using the Montgomery–Åsberg Depression Rating Scale (MADRS), a 10-item clinician-rated scale designed to assess depressive symptom severity and changes over treatment, with total scores ranging from 0 to 60 and higher scores indicating greater symptom severity [52]. Baseline MADRS scores ranged from 9 to 17. We additionally report the Behavioral Inhibition System/Behavioral Activation System (BIS/BAS) scales, which assess dispositional sensitivity to behavioral inhibition and activation [53]. The BAS measure comprises Drive, Fun Seeking, and Reward Responsiveness subscales. Table A5 also reports participant-specific observation durations and the completeness of self-report and passive sensing measures prior to imputation.

For each participant, we examine daily self-reported stress together with three passive sensing variables: daily step count, conversation time, and sleep duration. These variables represent different behavioral domains, namely physical activity, sociability, and sleep behavior, which have been associated with stress [54,55,56]. Participants reported their perceived stress daily in response to the question, “Do you feel stressed today?” A fixed time of day for administering this daily assessment was not specified in the available study documentation. Responses were recorded using five response categories on a 1–5 scale: 1 = Not at all, 2 = Slightly, 3 = Moderately, 4 = Very much, and 5 = Extremely. We therefore refer to this measure throughout the manuscript as daily self-reported stress. Passive sensing data were collected through the Measure smartphone application using the HealthRhythms platform [51]. Daily step count was obtained from the smartphone pedometer. Conversation time was derived from the VoicePresence stream and represents the percentage of voice-sensing time during which voice was detected, calculated as a duration-weighted average of the voiced percentages across sensing intervals. Sleep duration was an inferred measure derived from Activity/Pedometer data and represents the estimated number of seconds slept per day. The passive sensing variables were summarized daily to match the observation frequency of the self-reported stress measure. Since the passive sensing variables are measured on different scales, we analyze each variable in its original units without normalization. Information on medication or treatment changes and independently documented clinical events during the observation period was not available in the analytic data used for this study. Although MADRS assessments were available at scheduled study visits, these assessments were not treated as event-level ground truth for validating the identified hotspots. Accordingly, the real data application is used as a methodological illustration, and detected hotspots are not interpreted as independently validated clinical events.

**Figure 2:**
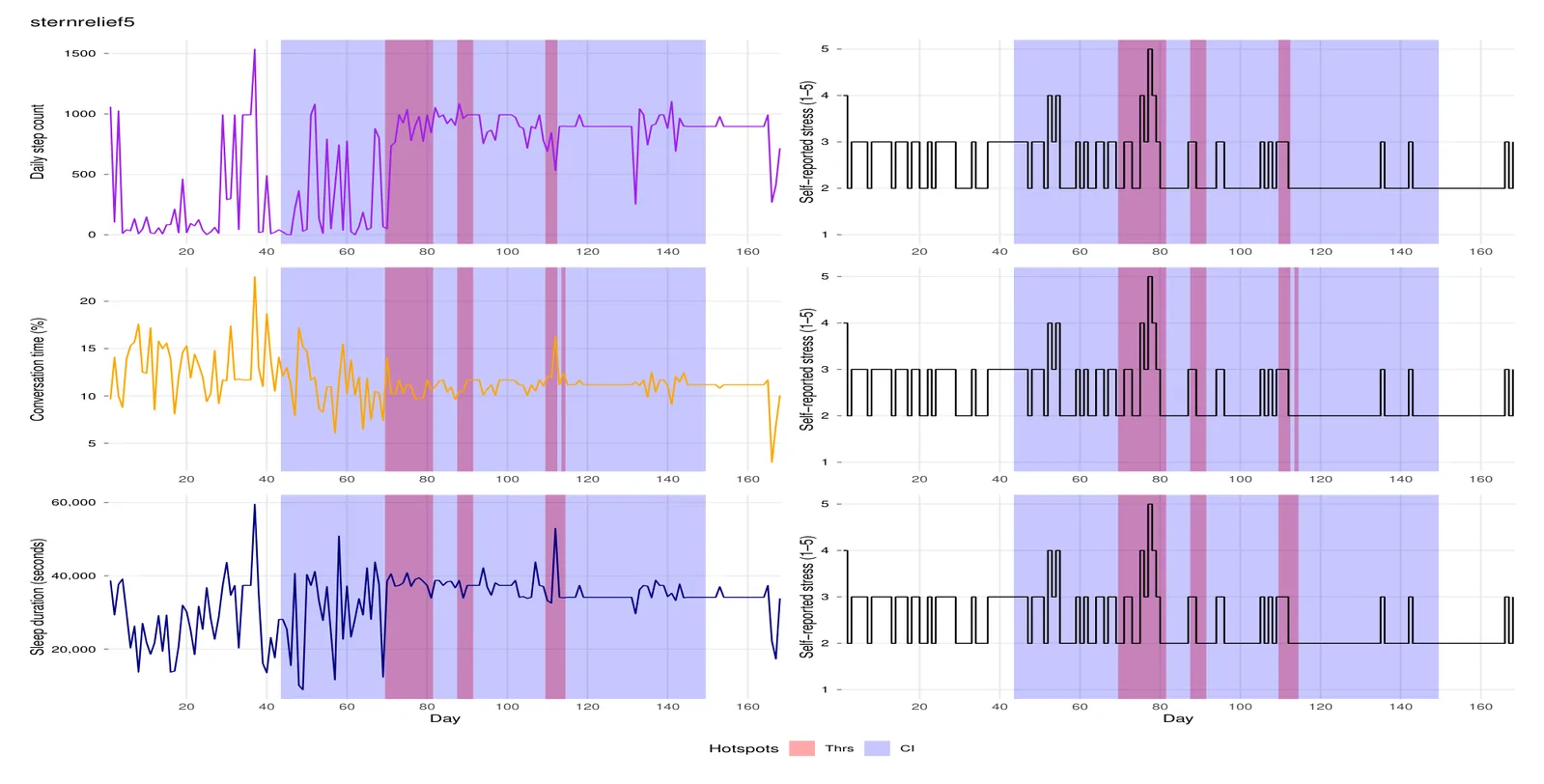
Identified hotspots for sternrelief5 with G=40 across three combinations of passive sensing variables (**left**; daily step count, conversation time, and sleep duration in each row) and daily self-reported stress (**right**). The thresholding and confidence-interval rules are shown as red and blue shaded areas, respectively.

Since the ground-truth locations of distributional changes are unknown in this real data application, our objective is not to assess detection accuracy but to illustrate how the proposed procedures summarize temporally proximate distributional changes in daily self-reported stress and passive sensing variables. We present sternrelief5 with G=40 as a representative example in Figure 2. For completeness, Table A6 in Appendix A.3 reports the estimated change points, identified hotspots, and stress-only change point support for all four participants, passive sensing variables, and window sizes. The corresponding visualizations for sternrelief15, sternrelief16, and sternrelief6 are also provided in Appendix A.3.

Figure 2 illustrates the hotspot regions identified for sternrelief5 with G=40. Joint-MOSUM applied to self-reported stress alone identifies change points at days 79 and 111. The thresholding rule identifies relatively localized hotspots around days 70–81, 88–91, and 110–114, with slight variations across the three passive sensing variables, covering both stress-only change points. In contrast, the CI rule produces a substantially broader interval, [44,149], for all three variables. Across the four participants, the identified hotspots vary with the passive sensing variable and window size, although overlapping regions are observed across different behavioral measures in several instances. These findings illustrate how the two rules provide different representations of potential temporal concordance between self-reported stress and passive sensing variables.

We conducted two supplementary analyses to further examine the relationship between stress-only change point detection and paired hotspot construction. First, Table A6 in Appendix A.3 compares the stress-only change points obtained by applying Joint-MOSUM to daily self-reported stress (the *Y* column) with those obtained from the four cross-series Bi-MOSUM detectors (YX, $YX^{2}$, $Y^{2}X$, and $Y^{2}X^{2}$). We additionally report the proportion of stress-only change points contained in the corresponding paired hotspots under each construction rule. These results illustrate how estimated change point locations vary across detectors and the extent to which stress-only changes are retained in the resulting hotspot regions.

Second, we conducted a temporal-shift negative-control analysis to assess whether the paired hotspots depend on the observed temporal alignment between daily self-reported stress and the passive sensing series. For each participant, passive sensing variable, and window size, the stress series was held fixed while the passive sensing series was circularly shifted over all nonzero temporal offsets. The thresholding hotspot rule was reapplied to each shifted alignment, and stress-only change point (CP) support and hotspot coverage were compared with those under the observed alignment. Table A7 in Appendix A.3 reports the median, interquartile range, and exceedance proportion across shifted alignments. For sternrelief5 with G=40 and sternrelief6 with both window sizes, CP support remains unchanged across shifts. In contrast, for sternrelief15 with G=40, the observed CP support exceeds that of many shifted alignments for step count and sleep duration, with exceedance proportions of 0.221 and 0.166, respectively. Moreover, for sternrelief16 with G=40, conversation time exhibits unchanged CP support but higher hotspot coverage under the observed alignment than under all shifted alignments. Overall, these results indicate that sensitivity to temporal alignment varies across participants, passive sensing variables, and window sizes, and that hotspot coverage provides information beyond CP support.

## 6. Discussion

In this article, we developed a framework for identifying candidate temporal hotspots that may reflect distributional changes in self-reported stress and passive sensing variables occurring at the same or nearby times. The proposed approach is intended as an auxiliary tool for examining temporal concordance between self-reported stress and behavioral measures derived from passive sensing data. Building on MOSUM-type CPD methods, the Joint- and Bi-MOSUM procedures detect changes in statistical features within and across series, and the resulting change point information is combined through thresholding and confidence-interval rules to construct hotspot regions. In this way, the framework summarizes potentially asynchronous distributional changes as interpretable temporal regions rather than isolated time points.

The simulation studies examined both change point detection and hotspot construction. The Joint- and Bi-MOSUM procedures achieved competitive detection rates across a range of distributional-change settings, with performance depending on the window size and the number of change points. However, the improved detection rates in several settings were accompanied by higher false positive rates under the no-change scenario. The two hotspot construction rules exhibited different trade-offs between retaining true change locations and producing localized temporal intervals. The sensitivity analyses showed relatively high hit rates under the examined levels of temporal dependence and moderate to high agreement in identified hotspots across repeated randomized Likert transformations. The latter analysis also indicated greater stability for the thresholding rule and a larger window size. In the real-data application, hotspot regions varied across passive sensing variables and window sizes, with overlapping regions observed in several instances. The temporal-shift analysis also revealed varying sensitivity to temporal alignment across participants and sensing variables, highlighting the exploratory nature of the identified hotspots.

Several limitations suggest directions for future work. First, the stress measure analyzed in this study is based on a subjective, single-item daily self-report and should not be interpreted as equivalent to a validated psychiatric symptom scale, a physiological measure of stress, depressive symptom severity, or clinician-rated deterioration. A hotspot may reflect an increase or decrease in self-reported stress, a change in its variability, or a combination of these changes, and therefore does not necessarily indicate worsening stress or a clinically meaningful stress episode. Rather, identified hotspots should be interpreted as periods warranting further examination. Accordingly, the four-participant application is intended as a methodological illustration rather than a clinical validation of the proposed framework. Establishing clinical relevance would require comparison with independent assessments of stressful episodes or other external clinical criteria.

Second, hotspot intervals can be relatively broad when uncertainty in change point locations or temporal asynchrony is substantial and should therefore be interpreted as approximate temporal regions rather than precise event times. Third, the proposed procedure is retrospective rather than a real-time detection method because the MOSUM statistics use observations on both sides of a candidate time point. With window sizes of G=20 and G=40, identifying a change may require up to 20 or 40 subsequent daily observations, respectively. Thus, the current framework is designed for retrospective identification of temporal regions of distributional change rather than immediate intervention. Although the AR(1) sensitivity analysis provides empirical evidence under the examined levels of temporal dependence, the threshold calibration and bootstrap procedures were not adjusted for serial dependence, and their theoretical properties under more general dependence structures remain to be investigated.

Finally, the present study is limited to smartphone-based behavioral sensing measures, as physiological data from wearable devices were not available. Consequently, we could not evaluate whether the proposed framework identifies similar or complementary hotspots using physiological measures. Future studies incorporating both behavioral and physiological sensing data could examine the consistency and complementary contributions of different sensing modalities to hotspot identification. Smartphone-derived behavioral measures can be collected using widely available devices but may be affected by device nonuse and missingness, whereas wearable sensors may provide more continuous physiological measurements when worn consistently. As physiological sensing data were unavailable and our framework does not directly classify stress states, we could not assess performance trade-offs relative to physiological stress-detection approaches.

## Figures and Tables

**Table 1:** Change point detection performance for the univariate simulations over 500 replications. DR(5) measures complete recovery of true change points within five time points. NMR(5) measures the proportion of replications in which no estimated change point matches any true change point within five time points, conditional on at least one estimated change point. Higher DR and lower NMR indicate better performance under their respective criteria; the best value in each case is shown in bold.

	DR(5)						NMR(5)					
# of change points: 1	Case 1	Case 2	Case 3	Case 4	Case 5	Case 6	Case 1	Case 2	Case 3	Case 4	Case 5	Case 6
Joint-MOSUM (G=20)	0.9300	0.8520	0.7740	0.8280	0.7380	0.5520	0.0190	**0.0427**	0.0675	0.0612	0.0911	0.1636
Joint-MOSUM (G=40)	**0.9400**	**0.8980**	**0.8060**	**0.8740**	**0.8340**	**0.6420**	0.0408	0.0587	0.0944	0.0761	0.1013	0.2152
PELT	0.9360	0.8700	0.7480	0.8240	0.7640	0.5340	**0.0147**	0.0481	0.0483	0.0485	**0.0521**	0.1159
stepR	0.9100	0.7880	0.7260	0.7760	0.6600	0.5160	0.0236	0.0837	0.0644	0.0628	0.1176	0.1042
WBS	0.9240	0.8020	0.7740	0.8020	0.7120	0.5880	0.0294	0.0782	0.1103	0.0866	0.1442	0.1833
MOSUM (G=20)	0.9020	0.7380	0.7280	0.7720	0.6060	0.4900	0.0217	0.0514	**0.0396**	**0.0326**	0.0762	**0.0926**
MOSUM (G=40)	0.9140	0.7820	0.7640	0.8060	0.6680	0.5460	0.0399	0.0735	0.0683	0.0606	0.1141	0.1727
# of change points: 2	Case 1	Case 2	Case 3	Case 4	Case 5	Case 6	Case 1	Case 2	Case 3	Case 4	Case 5	Case 6
Joint-MOSUM (G=20)	0.7840	**0.6900**	**0.5660**	**0.6720**	**0.5180**	**0.3040**	0.0060	0.0141	0.0125	**0.0081**	**0.0315**	0.0618
Joint-MOSUM (G=40)	0.3740	0.3120	0.2780	0.3380	0.2420	0.1480	0.0420	0.0265	0.0370	0.0161	0.0458	0.0951
PELT	0.8140	0.6840	0.4660	0.6260	0.4700	0.1380	0.0040	**0.0104**	0.0174	0.0124	0.0419	0.0932
stepR	0.7460	0.5880	0.4140	0.5340	0.3200	0.1040	0.0041	0.0333	0.0491	0.0367	0.0716	0.0863
WBS	**0.8260**	0.6760	**0.5660**	0.6700	0.4540	0.2800	**0.0020**	0.0225	0.0332	0.0142	0.0726	0.0780
MOSUM (G=20)	0.7640	0.5980	0.5080	0.5960	0.3600	0.2220	**0.0020**	0.0165	**0.0108**	0.0083	0.0371	**0.0517**
MOSUM (G=40)	0.1100	0.1020	0.0780	0.1080	0.0360	0.0200	0.0060	0.0289	0.0383	0.0267	0.0680	0.1053
# of change points: 3	Case 1	Case 2	Case 3	Case 4	Case 5	Case 6	Case 1	Case 2	Case 3	Case 4	Case 5	Case 6
Joint-MOSUM (G=20)	**0.7520**	**0.6600**	**0.4440**	**0.5800**	**0.4500**	**0.1940**	**0.0000**	**0.0000**	0.0060	**0.0020**	**0.0061**	0.0324
Joint-MOSUM (G=40)	0.1560	0.0920	0.0500	0.0680	0.0340	0.0040	0.0180	0.0240	0.0610	0.0362	0.0917	0.1794
PELT	0.6760	0.5600	0.2720	0.4240	0.2740	0.0580	**0.0000**	0.0060	0.0125	0.0081	0.0211	0.0796
stepR	0.6240	0.4200	0.1960	0.3380	0.1860	0.0440	0.0040	0.0120	0.0288	0.0304	0.0503	0.0869
WBS	0.7100	0.5600	0.3820	0.4980	0.2980	0.1600	0.0020	0.0080	0.0061	0.0060	0.0307	0.0568
MOSUM (G=20)	0.6800	0.5040	0.3780	0.4360	0.2420	0.1420	0.0020	0.0020	**0.0041**	0.0040	0.0127	**0.0317**
MOSUM (G=40)	0.0000	0.0040	0.0000	0.0020	0.0040	0.0040	0.0160	0.0202	0.0349	0.0449	0.0857	0.1199

**Table 2:** Change point detection performance for the bivariate simulations over 500 replications. DR(5) measures complete recovery of the pooled true change points within five time points. NMR(0) measures the proportion of replications in which no estimated change point exactly matches any true change point, conditional on at least one estimated change point. Higher DR and lower NMR indicate better performance under their respective criteria; the best value in each case is shown in bold.

	DR(5)						NMR(0)					
# of change points: (1, 1)	Case 1	Case 2	Case 3	Case 4	Case 5	Case 6	Case 1	Case 2	Case 3	Case 4	Case 5	Case 6
Bi-MOSUM (G=20)	0.8180	0.7280	0.6060	0.6360	0.5760	0.3880	0.0380	0.1225	0.1639	0.1303	0.2973	0.4504
Bi-MOSUM (G=40)	**0.9600**	**0.9120**	**0.8660**	**0.8860**	**0.7680**	**0.6320**	**0.0180**	**0.0480**	**0.0848**	**0.0541**	**0.1469**	**0.2454**
ecp	0.7780	0.5940	0.5420	0.5500	0.3940	0.2820	0.0421	0.1356	0.1843	0.1520	0.3043	0.4725
SBS	0.1420	0.1320	0.0740	0.1140	0.0960	0.0280	0.1288	0.2624	0.3478	0.2926	0.4155	0.5756
DCBS	0.8180	0.6880	0.5300	0.5840	0.4760	0.2580	0.0602	0.1455	0.1967	0.1527	0.3347	0.4911
NMP	0.7500	0.6940	0.6380	0.5820	0.5260	0.4680	0.0661	0.1804	0.2325	0.2184	0.3896	0.4960
# of change points: (1, 2)	Case 1	Case 2	Case 3	Case 4	Case 5	Case 6	Case 1	Case 2	Case 3	Case 4	Case 5	Case 6
Bi-MOSUM (G=20)	**0.9160**	**0.7880**	**0.6720**	**0.7980**	**0.5940**	**0.3700**	**0.0040**	0.0381	0.0863	0.0482	0.1556	0.3080
Bi-MOSUM (G=40)	0.6680	0.5460	0.5240	0.5820	0.3880	0.2800	**0.0040**	**0.0160**	**0.0560**	**0.0200**	**0.0942**	**0.1822**
ecp	0.5920	0.3220	0.2420	0.3220	0.1120	0.0720	0.0220	0.1002	0.1723	0.0884	0.2787	0.4250
SBS	0.0000	0.0080	0.0000	0.0040	0.0100	0.0000	0.1354	0.2620	0.3769	0.2563	0.4565	0.5462
DCBS	0.3740	0.3240	0.1720	0.2660	0.1960	0.0480	0.0480	0.1062	0.1956	0.1245	0.3074	0.4657
NMP	0.2440	0.1760	0.1640	0.1860	0.0920	0.0860	0.0480	0.1343	0.1720	0.1420	0.3133	0.4415
# of change points: (2, 2)	Case 1	Case 2	Case 3	Case 4	Case 5	Case 6	Case 1	Case 2	Case 3	Case 4	Case 5	Case 6
Bi-MOSUM (G=20)	**0.9240**	**0.7760**	**0.6720**	**0.7260**	**0.4940**	**0.2640**	**0.0020**	0.0160	0.0360	**0.0141**	0.0780	0.2231
Bi-MOSUM (G=40)	0.0180	0.0320	0.0200	0.0220	0.0440	0.0260	0.0040	**0.0040**	**0.0260**	0.0180	**0.0580**	**0.1457**
ecp	0.4620	0.2380	0.1880	0.2200	0.0700	0.0540	0.0080	0.0500	0.0920	0.0500	0.1760	0.3129
SBS	0.0000	0.0020	0.0000	0.0020	0.0020	0.0000	0.0711	0.1396	0.2274	0.1980	0.3756	0.4638
DCBS	0.2540	0.1520	0.0780	0.1020	0.0680	0.0160	0.0180	0.0542	0.1062	0.0842	0.2036	0.3739
NMP	0.0040	0.0080	0.0080	0.0020	0.0080	0.0040	0.0301	0.0640	0.1060	0.1060	0.2380	0.3534

**Table 3:** Hotspot construction performance over 200 replications for the thresholding (Thrs) and confidence-interval (CI) rules. The hit rate is the proportion of true change points in $\left\{Y_{t}\right\}$ contained in the estimated hotspots. Hotspot length is the length of the connected hotspot interval containing each true change point; a value of n=100 is assigned when the true change point is not contained in any estimated hotspot. Higher hit rates and shorter average hotspot lengths indicate better performance under these criteria, with the latter also reflecting penalties for missed detections.

	Hit Rate						Hotspot Length					
# of change points: 1, δ=0	Case 1	Case 2	Case 3	Case 4	Case 5	Case 6	Case 1	Case 2	Case 3	Case 4	Case 5	Case 6
Thrs (G=20)	0.8700	0.8600	0.8050	0.7750	0.6450	0.5550	36.4450	32.5200	35.7000	39.5950	46.0950	52.0100
Thrs (G=40)	0.9450	0.9300	0.8650	0.8950	0.8600	0.7550	59.1200	51.9900	52.0550	52.4500	44.5000	47.6000
CI (G=20)	0.8850	0.8750	0.8300	0.8000	0.7500	0.6400	18.2050	23.2750	27.2000	28.4600	36.8100	47.3050
CI (G=40)	0.9200	0.9000	0.8600	0.9100	0.8750	0.7700	16.2800	19.6900	25.1550	19.5250	27.8200	40.8100
# of change points: 1, δ=5	Case 1	Case 2	Case 3	Case 4	Case 5	Case 6	Case 1	Case 2	Case 3	Case 4	Case 5	Case 6
Thrs (G=20)	0.9100	0.8400	0.7750	0.7850	0.7250	0.5350	32.6700	34.6800	39.7400	37.5550	38.6050	52.5000
Thrs (G=40)	0.9750	0.9250	0.9000	0.9050	0.9000	0.7200	58.0500	53.9400	51.6500	51.5500	43.0450	51.3950
CI (G=20)	0.9300	0.8550	0.8000	0.8050	0.8000	0.6400	13.7400	23.4100	28.1000	29.4950	32.4000	48.6900
CI (G=40)	0.9150	0.8800	0.8900	0.8950	0.8450	0.8100	16.1400	20.3750	23.1200	21.0600	29.5500	40.5250
# of change points: 2, δ=0	Case 1	Case 2	Case 3	Case 4	Case 5	Case 6	Case 1	Case 2	Case 3	Case 4	Case 5	Case 6
Thrs (G=20)	0.9175	0.8225	0.7550	0.7875	0.6825	0.5125	38.0275	36.7050	39.4675	38.9975	41.6675	54.9425
Thrs (G=40)	0.9875	0.9350	0.9200	0.9225	0.8050	0.7075	61.5650	52.6725	50.6675	55.8575	51.2875	50.7150
CI (G=20)	0.9175	0.8475	0.7850	0.8150	0.7450	0.6225	42.4375	48.1375	49.4475	49.2100	55.0250	60.7550
CI (G=40)	0.6925	0.6750	0.6200	0.6250	0.6025	0.6075	51.7300	52.0600	54.1500	52.9800	57.2400	60.3725
# of change points: 2, δ=5	Case 1	Case 2	Case 3	Case 4	Case 5	Case 6	Case 1	Case 2	Case 3	Case 4	Case 5	Case 6
Thrs (G=20)	0.9075	0.8025	0.7250	0.8450	0.6750	0.5450	35.7125	38.0350	43.0550	33.9925	42.6025	51.8475
Thrs (G=40)	0.9650	0.9350	0.8825	0.9175	0.7975	0.6425	62.6075	54.1825	53.1350	53.9025	52.1600	57.7950
CI (G=20)	0.8875	0.8100	0.7800	0.8550	0.7675	0.6325	43.4975	49.0550	51.5775	45.8300	51.8650	60.5075
CI (G=40)	0.3650	0.4350	0.4550	0.4225	0.5100	0.5050	72.6475	68.5725	68.8825	70.7175	68.6375	70.5825

**Figure A3:**
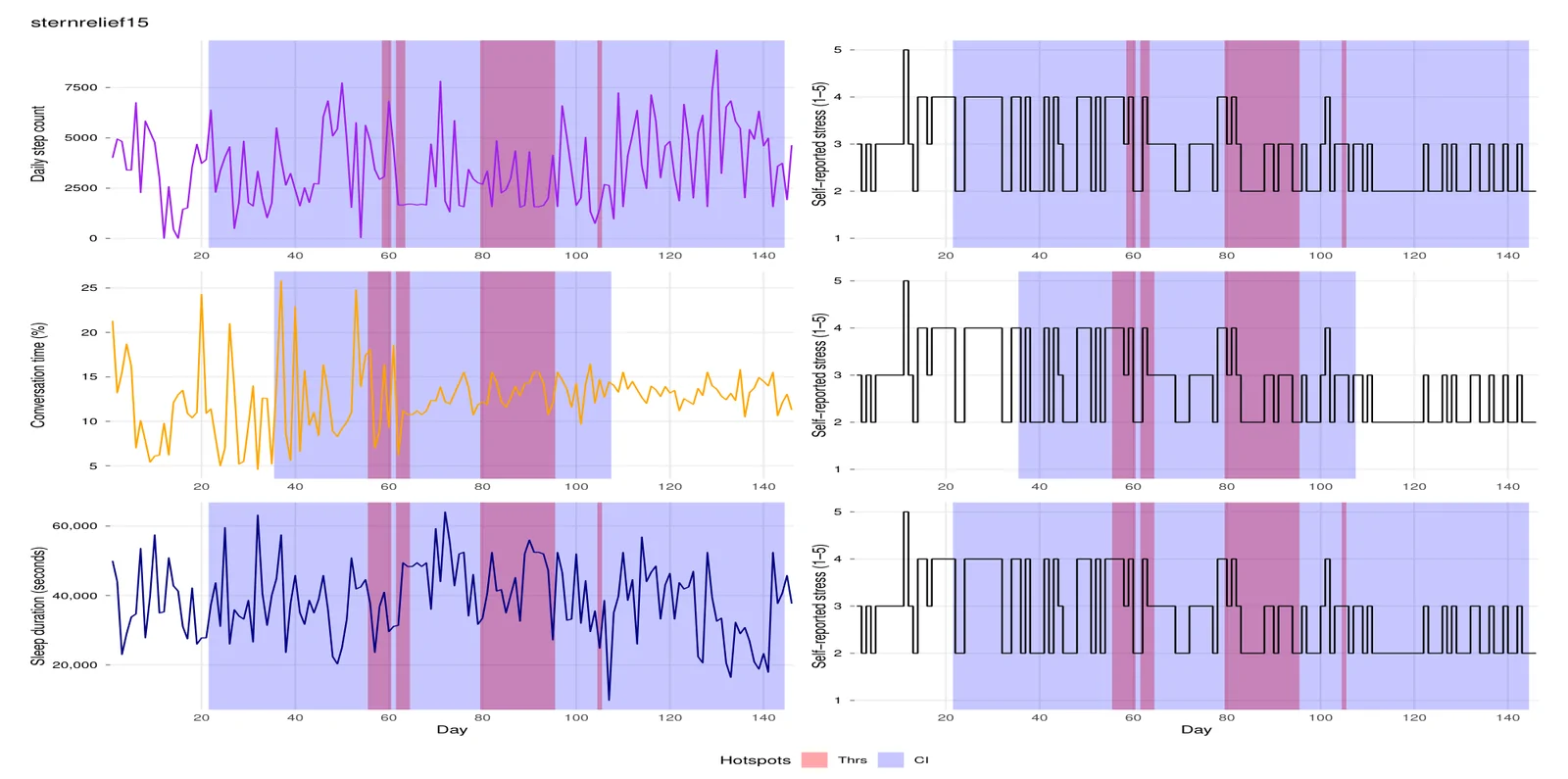
Identified hotspots for sternrelief15 with G=40 across three combinations of passive sensing variables ((**left**); daily step count, conversation time, and sleep duration in each row) and daily self-reported stress (**right**). The thresholding and confidence-interval rules are shown as red and blue shaded areas, respectively.

**Figure A4:**
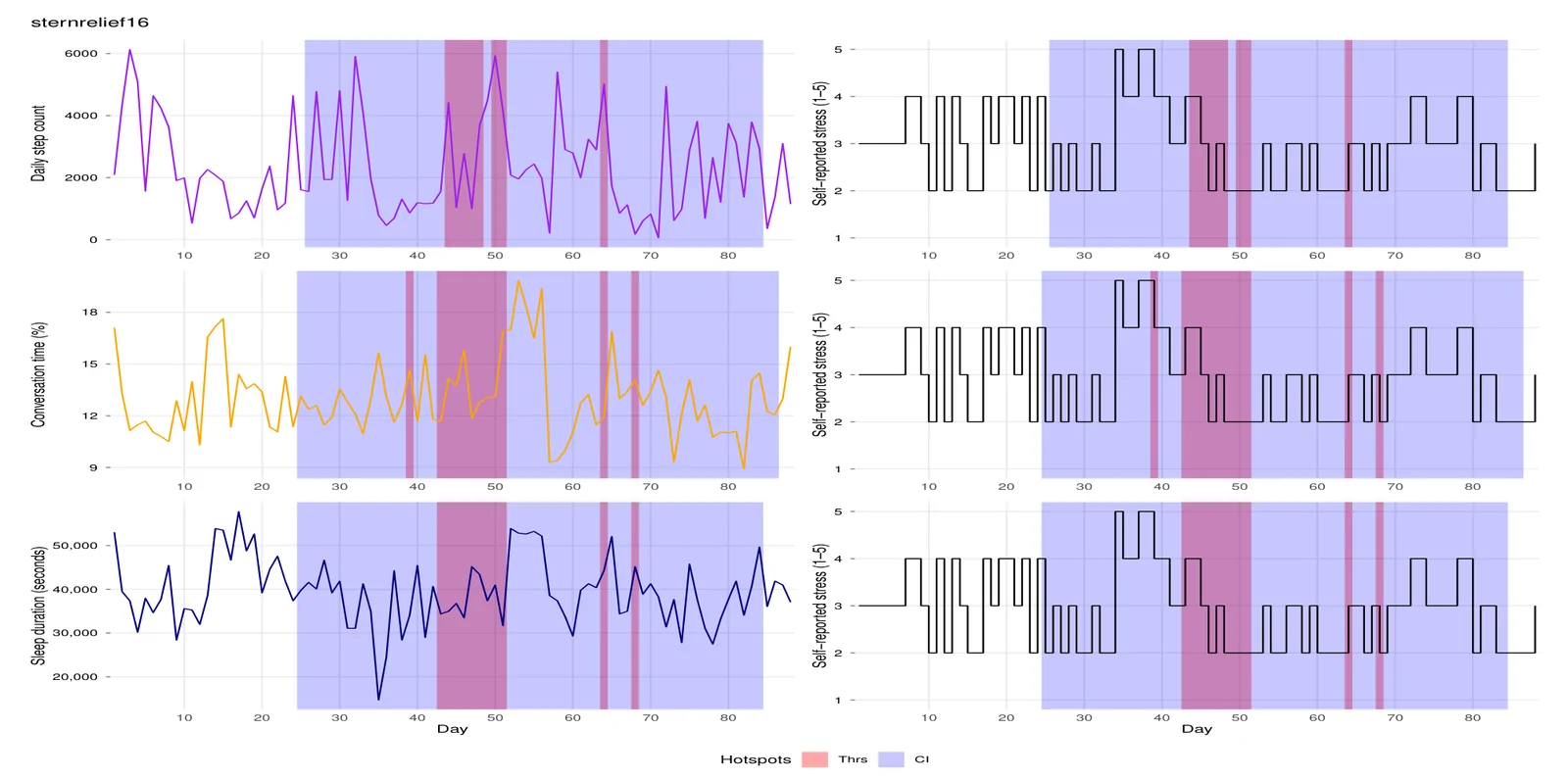
Identified hotspots for sternrelief16 with G=20 across three combinations of passive sensing variables ((**left**); daily step count, conversation time, and sleep duration in each row) and daily self-reported stress (**right**). The thresholding and confidence-interval rules are shown as red and blue shaded areas, respectively.

**Figure A5:**
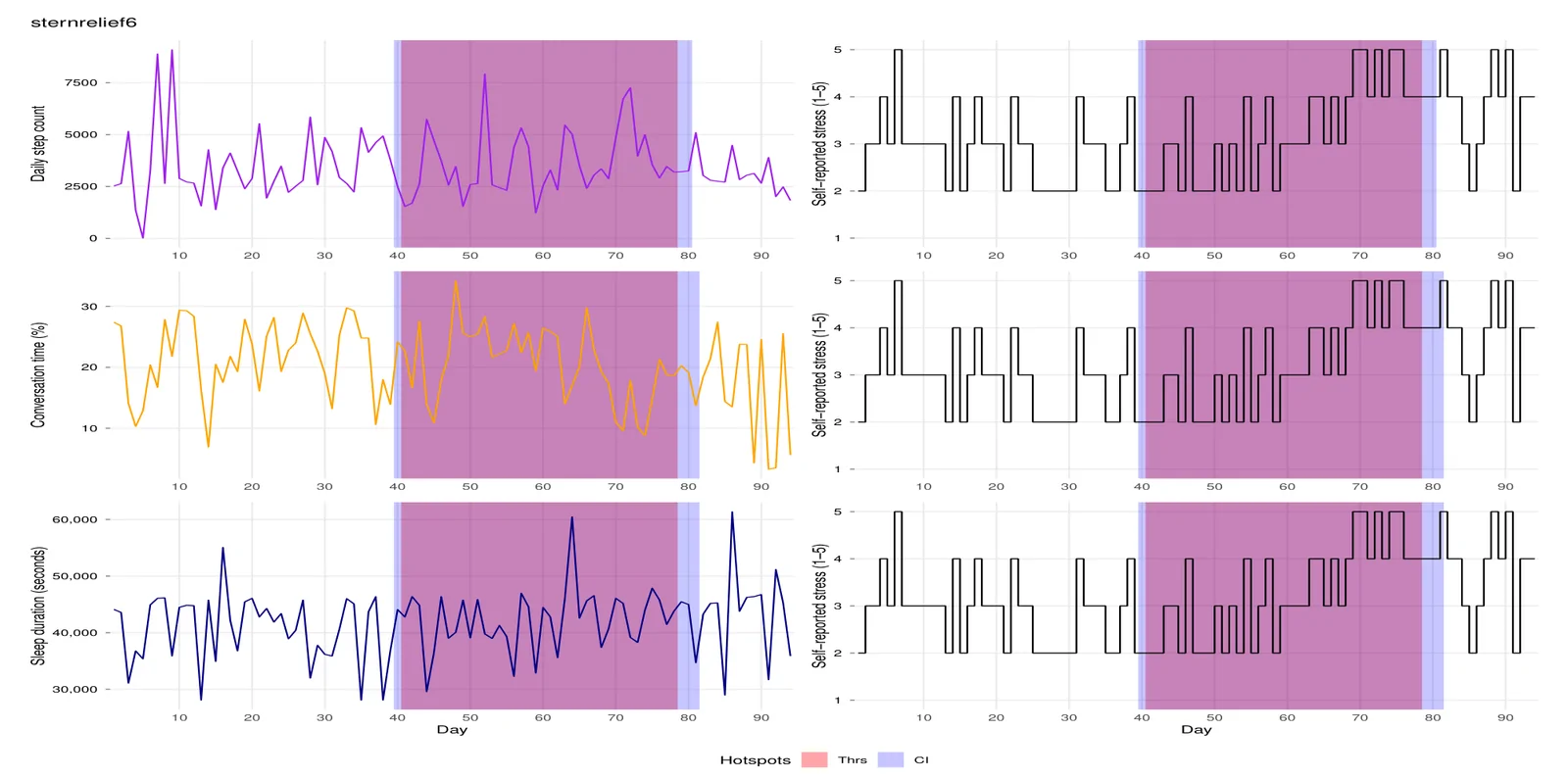
Identified hotspots for sternrelief6 with G=40 across three combinations of passive sensing variables ((**left**); daily step count, conversation time, and sleep duration in each row) and daily self-reported stress (**right**). The thresholding and confidence-interval rules are shown as red and blue shaded areas, respectively.

## Data Availability

The data presented in this study are available on request from the corresponding author due to privacy and ethical restrictions related to human participant data. The R code used for illustrating the method in Section 4.3 and for the simulation studies and data application in Section 5 is available on GitHub at https://github.com/yk748/hotspot (accessed on 22 October 2026).

## Abbreviations

The following abbreviations are used in this manuscript:

AR(1)	First-order autoregressive process
BAS	Behavioral Activation System
Bi-MOSUM	Bivariate moving sum
BIS	Behavioral Inhibition System
CDF	Cumulative distribution function
CPD	Change point detection
CI	Confidence interval
CT	Conversation time
CUSUM	Cumulative sum
DCBS	Double CUSUM binary segmentation
DR	Detection rate
FDR	False discovery rate
FHL	False hotspot length
FHR	False hotspot rate
FPR	False positive rate
Joint-MOSUM	Joint moving sum
MADRS	Montgomery–Åsberg Depression Rating Scale
MBIC	Modified Bayesian information criterion
mHealth	Mobile health
MHL	Mean hotspot length
ML	Machine learning
MOSUM	Moving sum
NHR	No-hotspot rate
NMP	Nonparametric method
NMR	No-match rate
PELT	Pruned exact linear time
SBS	Sparsified binary segmentation
SC	Step count
SD	Sleep duration
Thrs	Thresholding rule
WBS	Wild binary segmentation

## Appendix A. Additional Figures and Tables

### Appendix A.2. Additional Tables from Section 5.1, Section 5.2 and Section 5.3

**Table A1:** False positive rates (FPRs) for Joint-MOSUM and benchmark methods under the no-change scenario. The FPR is defined as the proportion of the 500 replications in which at least one change point is detected when no true change point is present. A significance level of α=0.05 is used where applicable. Lower values indicate better control of spurious detections.

Method	FPR
Joint-MOSUM (G=20)	0.172
Joint-MOSUM (G=40)	0.112
PELT	0.046
stepR	0.020
WBS	0.070
MOSUM (G=20)	0.046
MOSUM (G=40)	0.024

**Table A2:** Hotspot behavior over 200 replications for the thresholding (Thrs) and confidence-interval (CI) rules. When neither series changes or only $\left\{X_{t}\right\}$ changes, FHR is the proportion of replications with at least one estimated hotspot, and FHL is the average total length of the estimated hotspots (zero if none are detected). When only $\left\{Y_{t}\right\}$ changes, NHR is the proportion of replications with no estimated hotspot, and MHL is the average total length of the estimated hotspots (including zero lengths). Lower FHR and FHL indicate fewer spurious hotspots in the first two scenarios; lower NHR indicates fewer empty hotspot sets in the *Y*-only scenario. NHR does not measure whether a hotspot contains the true change point.

	Rate (FHR/NHR)			Length (FHL/MHL)		
No change in either series (FHR/FHL)						
Thrs (G=20)	0.1350			0.2850		
Thrs (G=40)	0.1600			0.7200		
CI (G=20)	0.1450			4.3850		
CI (G=40)	0.1550			6.2400		
Only $\left\{X_{t}\right\}$ changes (FHR/FHL)	Case 1	Case 2	Case 4	Case 1	Case 2	Case 4
Thrs (G=20)	0.1700	0.1700	0.1800	0.4550	0.3350	0.4050
Thrs (G=40)	0.2100	0.1600	0.1800	1.0200	0.4600	0.7850
CI (G=20)	0.2250	0.1900	0.2200	6.3100	5.6700	6.2200
CI (G=40)	0.2150	0.1500	0.1850	8.7150	5.2850	6.2300
Only $\left\{Y_{t}\right\}$ changes (NHR/MHL)	Case 1	Case 2	Case 4	Case 1	Case 2	Case 4
Thrs (G=20)	0.0750	0.1500	0.1500	23.0500	16.9300	16.9100
Thrs (G=40)	0.0150	0.0650	0.0500	52.6350	40.9550	40.4250
CI (G=20)	0.0800	0.1450	0.1400	7.9800	10.7850	11.8900
CI (G=40)	0.0150	0.0650	0.0500	10.0450	14.8800	14.6050

**Table A3:** Hotspot performance under temporal dependence over 200 replications. The errors in both series follow stationary AR(1) processes with autocorrelation parameter ρ. The hit rate is the proportion of true change points in $\left\{Y_{t}\right\}$ contained in the estimated hotspots, and hotspot length is the length of the connected hotspot interval associated with each true change point, with a value of n=100 assigned when the true change point is not contained in an estimated hotspot. Higher hit rates and shorter hotspot lengths indicate better performance.

	Hit Rate			Hotspot Length		
ρ=0.3, δ=0	Case 1	Case 2	Case 4	Case 1	Case 2	Case 4
Thrs (G=20)	0.8950	0.8300	0.8200	33.5200	35.8250	35.6650
Thrs (G=40)	0.9300	0.9150	0.8600	58.6900	51.8900	53.7150
CI (G=20)	0.8650	0.8400	0.8600	23.8750	29.4050	26.7600
CI (G=40)	0.8800	0.9150	0.8950	24.0150	22.5400	25.2900
ρ=0.3, δ=5	Case 1	Case 2	Case 4	Case 1	Case 2	Case 4
Thrs (G=20)	0.9050	0.8700	0.8250	33.4250	32.0800	34.7500
Thrs (G=40)	0.9450	0.9450	0.9400	60.5950	51.7750	47.6800
CI (G=20)	0.8850	0.8800	0.8200	19.9450	24.6050	33.2100
CI (G=40)	0.9200	0.8550	0.8850	17.4100	26.6750	28.6850
ρ=0.6, δ=0	Case 1	Case 2	Case 4	Case 1	Case 2	Case 4
Thrs (G=20)	0.9200	0.8700	0.8500	34.7700	33.8200	35.1600
Thrs (G=40)	0.9800	0.9450	0.9250	60.5100	51.4850	52.8650
CI (G=20)	0.8550	0.8250	0.9000	30.3300	36.3300	37.1350
CI (G=40)	0.8550	0.9000	0.8750	29.3350	34.3100	35.9300
ρ=0.6, δ=5	Case 1	Case 2	Case 4	Case 1	Case 2	Case 4
Thrs (G=20)	0.9350	0.8400	0.8550	32.2600	37.0350	35.3600
Thrs (G=40)	0.9800	0.9250	0.9300	60.4500	56.4750	54.1050
CI (G=20)	0.8750	0.8750	0.8800	25.8750	37.0300	34.4550
CI (G=40)	0.8750	0.8450	0.8400	26.1150	36.3700	34.9000

**Table A4:** Sensitivity of hotspot identification to the random draws used in the randomized Likert transformation. For each simulated dataset, the ordinal series is held fixed and the transformation in (1) is repeated M=10 times using independent random draws. The reported values are the average pairwise Jaccard similarities among the resulting hotspot sets, averaged across simulation replications. Values closer to one indicate greater stability with respect to the random draws.

	Average Jaccard Similarity		
δ=0	Case 1	Case 2	Case 4
Thrs (G=20)	0.7311	0.7310	0.6966
Thrs (G=40)	0.7680	0.7720	0.7558
CI (G=20)	0.6386	0.6273	0.6327
CI (G=40)	0.6492	0.6444	0.6408
δ=5	Case 1	Case 2	Case 4
Thrs (G=20)	0.7087	0.6980	0.6759
Thrs (G=40)	0.7753	0.7595	0.7840
CI (G=20)	0.6233	0.6443	0.6389
CI (G=40)	0.6676	0.6554	0.6581

### Appendix A.3. Additional Tables and Figures from Section 5.4

**Table A5:** Demographic and clinical characteristics, observation durations, and data completeness of the four participants included in the data application.

Participant	Age	Gender	MADRS	BAS Drive	BAS Fun	BAS Reward	BIS	Days	Self-Report	Steps	Conversation	Sleep
	(Years)		Total		Seeking	Responsiveness			(%)	(%)	(%)	(%)
sternrelief5	80.4	Female	13	8	12	18	20	168	47.6	22.6	35.7	29.8
sternrelief6	60.2	Female	17	13	13	17	18	94	97.9	55.3	56.4	58.5
sternrelief15	82.5	Female	15	11	12	16	21	146	34.2	82.2	39.7	82.9
sternrelief16	66.4	Female	9	11	14	19	26	88	89.8	89.8	27.0	89.8

MADRS = Montgomery–Åsberg Depression Rating Scale; BAS = Behavioral Activation System; BIS = Behavioral Inhibition System. MADRS and BIS/BAS values shown are baseline scores. Data completeness percentages are calculated prior to imputation.

**Table A6:** Estimated change points and identified hotspots in the real-data application. The five change point columns report the stress-only Joint-MOSUM estimates (*Y*) and the four cross-series Bi-MOSUM estimates (YX, $YX^{2}$, $Y^{2}X$, and $Y^{2}X^{2}$). The hotspot columns report the thresholding (Thrs) and confidence-interval (CI) regions. Stress-only CP support is the number of stress-only change points contained in the paired hotspot divided by the total number of stress-only change points; – indicates that no stress-only change points were detected, or no estimated points/intervals for other columns. SC, CT, and SD denote daily step count, conversation time, and sleep duration, respectively. The significance level is α=0.05.

			Change Points					Hotspots		Stress-Only CP Support	
Participants	*G*	Variable	*Y*	YX	$YX^{2}$	$Y^{2}X$	$Y^{2}X^{2}$	Thrs	CI	Thrs	CI
sternrelief15	20	SC	–	–	–	–	–	–	–	–	–
		CT	–	–	–	–	–	–	–	–	–
		SD	–	{62}	–	{57,62,126}	–	–	–	–	–
	40	SC	{62,82,105}	{60,82,105}	{82,93}	{106,126}	–	{[59,60], [62,63], [80,95], [105,105]}	{[22,144]}	3/3	3/3
		CT	{62,82,105}	{82}	{62,82}	–	{62}	{[56,60], [62,64], [80,95]}	{[36,107]}	2/3	3/3
		SD	{62,82,105}	{62,101,126}	{89,105}	{62,101,126}	–	{[56,60], [62,64], [80,95], [105,105]}	{[22,144]}	3/3	3/3
sternrelief16	20	SC	{45,68}	{50,65}	{45,50}	{47}	–	{[44,48], [50,51], [64,64]}	{[26,84]}	1/2	2/2
		CT	{45,68}	{45,56,65}	{45,65}	{38,56}	{15,39,45,67}	{[39,39], [43,51], [64,64], [68,68]}	{[25,86]}	2/2	2/2
		SD	{45,68}	{30,45,65}	{47,65}	{30,46}	–	{[43,51], [64,64], [68,68]}	{[25,84]}	2/2	2/2
	40	SC	{44}	{45}	{45}	{47}	{4}	{[44,48]}	{[15,73]}	1/1	1/1
		CT	{44}	{45,57}	{45}	{57}	{48}	{[40,51]}	{[15,73]}	1/1	1/1
		SD	{44}	{45}	{45}	–	{46}	{[44,48]}	{[15,73]}	1/1	1/1
sternrelief5	20	SC	{1,113}	{72,79,166}	{78}	{72,111,166}	{78,111}	{[111,112], [114,114]}	{[96,130]}	0/2	1/2
		CT	{1,113}	{166}	{79,166}	{114,166}	{70,114,166}	{[111,114]}	{[96,130]}	1/2	1/2
		SD	{1,113}	{78,166}	{71,78,166}	{112,166}	{69,112,166}	{[111,114]}	{[96,130]}	1/2	1/2
	40	SC	{79,111}	{50,77,166}	{83,166}	{50,77,111,166}	{83,111,166}	{[70,81], [88,91], [110,112]}	{[44,149]}	2/2	2/2
		CT	{79,111}	{52,79,91,166}	{79,166}	{50,111,166}	{70,112,166}	{[70,81], [88,91],[110,112], [114,114]}	{[44,149]}	2/2	2/2
		SD	{79,111}	{49,78,112,166}	{78,166}	{49,72,112,166}	{69,112,166}	{[70,81],[88,91], [110,114]}	{[44,149]}	2/2	2/2
sternrelief6	20	SC	{22,62}	{22,62}	{22,62}	–	–	{[21,25], [51,67]}	{[3,41],[53,71]}	2/2	2/2
		CT	{22,62}	{22,45,62}	{22,62,89}	{66}	{89}	{[22,25], [50,67]}	{[3,41], [52,72]}	2/2	2/2
		SD	{22,62}	{24,62}	{22,62}	{63}	–	{[22,25], [51,65], [67,67]}	{[3,41], [52,72]}	2/2	2/2
	40	SC	{53,63}	{53,63}	{9,53,63}	–	{9}	{[41,78]}	{[40,80]}	2/2	2/2
		CT	{53,63}	{54,63,89}	{54,63,89}	{69,89}	{89}	{[41,78]}	{[40,81]}	2/2	2/2
		SD	{53,63}	{53,63}	{53,63}	–	–	{[41,78]}	{[40,81]}	2/2	2/2

**Table A7:** Temporal-shift negative-control analysis using all nonzero circular shifts of the passive sensing series. $S_{0}$ and $C_{0}$ denote stress-only change point support and the proportion of time points covered by thresholding hotspots under the observed alignment. Shifted columns report median (first–third quartile). $E_{\mathrm{shift}}$ and $E_{\mathrm{cov}}$ denote the proportions of shifted alignments with support and coverage at least as large as observed, respectively. SC, CT, and SD denote daily step count, conversation time, and sleep duration. – indicates undefined stress-only support when no stress-only change points are detected. These exceedance measures are descriptive, not calibrated *p*-values.

			CP Support			Hotspot Coverage		
Participant	*G*	Variable	Observed $S_{0}$	Shifted Median (IQR)	$E_{\mathrm{shift}}$	Observed $C_{0}$	Shifted Median (IQR)	$E_{\mathrm{cov}}$
sternrelief15	20	SC	–	–	–	0.000	0.000 (0.000–0.000)	1.000
		CT	–	–	–	0.000	0.000 (0.000–0.000)	1.000
		SD	–	–	–	0.000	0.000 (0.000–0.000)	1.000
	40	SC	1.000	0.667 (0.333–0.667)	0.221	0.144	0.116 (0.110–0.158)	0.290
		CT	0.667	1.000 (0.667–1.000)	1.000	0.164	0.151 (0.123–0.164)	0.407
		SD	1.000	0.667 (0.667–0.667)	0.166	0.171	0.116 (0.116–0.144)	0.055
sternrelief16	20	SC	0.500	0.500 (0.500–0.500)	1.000	0.091	0.080 (0.057–0.091)	0.172
		CT	1.000	1.000 (0.500–1.000)	0.517	0.136	0.125 (0.102–0.148)	0.264
		SD	1.000	0.500 (0.500–1.000)	0.460	0.125	0.125 (0.102–0.136)	0.517
	40	SC	1.000	0.000 (0.000–1.000)	0.460	0.057	0.023 (0.023–0.045)	0.034
		CT	1.000	1.000 (1.000–1.000)	1.000	0.136	0.114 (0.102–0.136)	0.000
		SD	1.000	1.000 (1.000–1.000)	0.943	0.057	0.057 (0.045–0.080)	0.379
sternrelief5	20	SC	0.000	0.500 (0.500–0.500)	1.000	0.018	0.024 (0.024–0.024)	1.000
		CT	0.500	0.500 (0.500–0.500)	0.934	0.024	0.024 (0.024–0.024)	0.060
		SD	0.500	0.500 (0.500–0.500)	0.916	0.024	0.024 (0.024–0.024)	0.030
	40	SC	1.000	1.000 (1.000–1.000)	1.000	0.113	0.113 (0.095–0.125)	0.443
		CT	1.000	1.000 (1.000–1.000)	1.000	0.119	0.113 (0.095–0.125)	0.311
		SD	1.000	1.000 (1.000–1.000)	1.000	0.125	0.125 (0.113–0.125)	0.605
sternrelief6	20	SC	1.000	1.000 (1.000–1.000)	1.000	0.234	0.234 (0.223–0.234)	0.022
		CT	1.000	1.000 (1.000–1.000)	1.000	0.234	0.234 (0.234–0.234)	0.204
		SD	1.000	1.000 (1.000–1.000)	1.000	0.213	0.223 (0.213–0.234)	0.946
	40	SC	1.000	1.000 (1.000–1.000)	1.000	0.404	0.394 (0.383–0.404)	0.194
		CT	1.000	1.000 (1.000–1.000)	1.000	0.404	0.415 (0.404–0.415)	0.548
		SD	1.000	1.000 (1.000–1.000)	1.000	0.404	0.394 (0.394–0.404)	0.215
